# The Pseudomonas aeruginosa sphBC genes are important for growth in the presence of sphingosine by promoting sphingosine metabolism

**DOI:** 10.1099/mic.0.001520

**Published:** 2025-01-10

**Authors:** Pauline DiGianivittorio, Lauren A. Hinkel, Jacob R. Mackinder, Kristin Schutz, Eric A. Klein, Matthew J Wargo

**Affiliations:** 1 Department of Microbiology and Molecular Genetics, Larner College of Medicine, University of Vermont, Burlington, USA; 2 Cellular, Molecular, and Biomedical Sciences Graduate Program, University of Vermont, Burlington, USA; 3 Biology Department, Rutgers University-Camden, Camden, USA

**Keywords:** lipid, pathogenesis, sphingosine

## Abstract

Sphingoid bases, including sphingosine, are important components of the antimicrobial barrier at epithelial surfaces where they can cause growth inhibition and killing of susceptible bacteria. *Pseudomonas aeruginosa* is a common opportunistic pathogen that is less susceptible to sphingosine than many Gram-negative bacteria. Here, we determined that the deletion of the *sphBCD* operon reduced growth in the presence of sphingosine. Using deletion mutants, complementation and growth assays in *P. aeruginosa* PAO1, we determined that the *sphC* and *sphB* genes, encoding a periplasmic oxidase and periplasmic cytochrome c, respectively, were important for growth on sphingosine, while *sphD* was dispensable under these conditions. Deletion of *sphBCD* in *P. aeruginosa* PA14, *Pseudomonas protegens* Pf-5 and *Pseudomonas fluorescens* Pf01 also showed reduced growth in the presence of sphingosine. The *P. aeruginosa sphBC* genes were also important for growth in the presence of two other sphingoid bases, phytosphingosine and sphinganine. In WT *P. aeruginosa*, sphingosine is metabolized to an unknown non-inhibitory product, as sphingosine concentrations drop in the culture. However, in the absence of *sphBC*, sphingosine accumulates, pointing to SphC and SphB as having a role in sphingosine metabolism. Finally, the metabolism of sphingosine by WT *P. aeruginosa* protected susceptible cells from full growth inhibition by sphingosine, pointing to a role for sphingosine metabolism as a public good. This work shows that the metabolism of sphingosine by *P. aeruginosa* presents a novel pathway by which bacteria can alter host-derived sphingolipids, but it remains an open question whether SphB and SphC act directly on sphingosine.

## Introduction

**Fig. 1.**
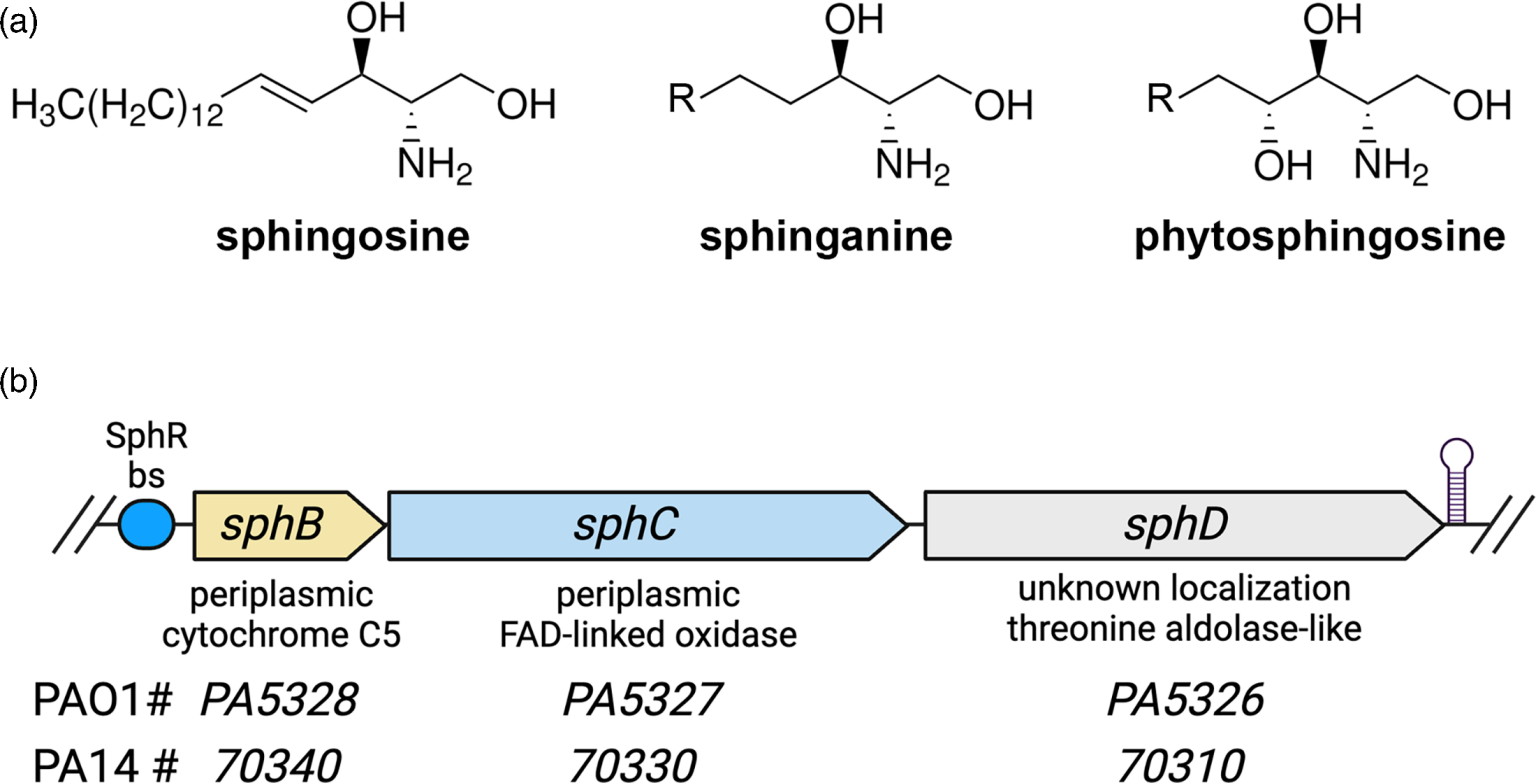
Sphingoid bases and arrangement of the *sphBCD* operon. (**a**) Structures of the sphingoid bases used in this study noting the head-group differences. All of the sphingoid bases used in this study are C18 versions, though there is tail length variation in naturally occurring versions from different body sites or organisms. (**b**) Organization of the *sphBCD* operon in *P. aeruginosa* showing the relative gene sizes, predicted functions and the gene numbers in the PAO1 and PA14 genomes. The SphR bs denotes the binding site for the sphingosine-responsive transcriptional activator SphR, and the hairpin at the right edge is the predicted rho-independent terminator.

In addition to their various cellular and signalling functions, some sphingolipids are key antimicrobial lipids with activity against both Gram-positive and Gram-negative bacteria [1,6]. Antimicrobial sphingolipids are found at sites throughout the body including the lungs, the skin and all mucosal surfaces [4,7,13]. Imbalances or deficiencies in barrier-associated sphingolipids, particularly sphingoid bases (examples in Fig. 1a), increase the chances of bacterial infection, illustrating the importance of these sphingolipids in defence against pathogens [14,16]. The initial antibacterial action for sphingoid bases is predicted to be bacterial membrane disruption, due to their amphiphilic and detergent-like properties, followed by the accumulation of sphingolipids in the cytosol, ultimately leading to cell death in both Gram-negative and Gram-positive bacteria [4,6, 17].

In Gram-negative bacteria, sphingolipid exposure causes the separation of the inner and outer membranes, similar to the type of damage caused by cationic antimicrobial peptides [6]. The concentrations of sphingoid bases needed to cause severe and cytotoxic membrane disruption in many bacteria are low, whereas *Serratia marcescens* and *Pseudomonas aeruginosa* are exceptions, requiring higher concentrations or specific media conditions. For example, the minimum bactericidal concentration for *P. aeruginosa* in most media is >1 mM, more than 300-fold higher than for *Staphylococcus aureus*, which often co-infect lungs and wounds [1]. However, *P. aeruginosa* killing can be seen with concentrations as low as 10 µM under distinct media and sphingoid base solubilization regimes [17], and sphingosine-dependent killing of *P. aeruginosa* also occurs intracellularly [18]. Although there are many factors that influence antimicrobial–bacterial interactions, the sphingolipid resistance profile of *P. aeruginosa* suggests that it possesses specific mechanisms for resistance to or detoxification of sphingoid bases.

*P. aeruginosa* is associated with a variety of infections, including hospital-acquired and ventilator-associated pneumonia and bacteraemia [19,22], as well as chronic lung infection in individuals with cystic fibrosis (CF) and chronic obstructive pulmonary disorder [22,28]. Many of these infection niches contain abundant sphingosine, other sphingoid bases and the sphingosine precursors sphingomyelin and ceramide [2,29,33], and a decrease in sphingosine concentration due to ceramide accumulation has been shown in CF [34,35]. Therapeutic intervention to treat ceramide accumulation can rescue the susceptibility of ceramide-accumulating animal models to *P. aeruginosa* infection [36]. Within the context of pulmonary infections, *P. aeruginosa*’s ability to detect sphingosine is correlated with a survival advantage [23].

Exposure of *P. aeruginosa* to pulmonary surfactant leads to the induction of a small set of sphingosine-responsive genes in an SphR-dependent manner, including a metabolic operon, *sphBCD,* encoding a predicted cytochrome c (SphB), predicted oxidoreductase enzyme (SphC) and predicted pyridoxal phosphate-dependent aldolase enzyme (SphD) [23] (Fig. 1b). We previously showed that the loss of *sphC* led to a small but statistically significant increase in *P. aeruginosa* death in the presence of sphingosine [23]. However, the conditions needed for sphingosine killing of *P. aeruginosa* are very specific, whereas *P. aeruginosa* exposure to sphingosine occurs in a variety of environments that do not mimic the media used in killing assays. Thus, based on their membership in the SphR regulon, we hypothesized that *sphBCD* primarily played a role in *P. aeruginosa* interaction with sphingosine independent of sphingosine killing. Here, we demonstrate that sphingosine can strongly suppress the growth of a *P. aeruginosa sphBCD* mutant, with follow-up experiments supporting the *sphBC* genes as important for *P. aeruginosa* growth in the presence of sphingosine via sphingosine detoxification. Sphingosine detoxification can function as a public good promoting the growth of sphingosine-susceptible *P. aeruginosa* mutants. Thus, in addition to understanding the role of the *sphBCD* genes, we also highlight the diversity of *P. aeruginosa* responses to sphingosine and that their responses are more complex than previously appreciated.

## Methods

### Strains and growth conditions

*P. aeruginosa* PA14, PAO1 and related mutant strains were maintained at 37 °C on *Pseudomonas* isolation agar (PIA) plates with 20 µg ml^−1^ gentamicin added when appropriate. *Pseudomonas fluorescens*, *Pseudomonas protegens* and strains of those species were maintained at 30 °C on lysogeny broth (LB) Lennox formulation plates. Prior to assay set-up, strains were grown shaking either at 37 or 30 °C overnight in a 1× MOPS medium [37], modified as previously described [38], and supplemented with 25 mM pyruvate and 5 mM glucose, adding in 20 µg ml^−1^ gentamicin when appropriate. For competition assays, *P. aeruginosa* PAO1 and *S. aureus* strains were maintained at 37 °C on LB plates. Prior to co-culture experiments, *P. aeruginosa* and *S. aureus* were grown shaking at 37 °C in 1× MOPS medium with 20 mM pyruvate and 5 mM glucose. All strains are listed in Table 1.

**Table 1.** Strain and construct list

Lab strain ID	Genotype	Plasmid	Source
MJ79	*P. aeruginosa* PAO1 WT	–	(PMID: 10984043) Stover *et al*.
LAH 83.2	∆*sphBCD* in PAO1	–	This study
PD47	∆*sphC* in PAO1	–	This study
PD132	*P. aeruginosa* PAO1 WT	pPD1	This study
PD136	∆*sphBCD* in PAO1	pPD1	This study
PD139	∆*sphBCD* in PAO1	pPD8	This study
PD121	∆*sphC* in PAO1	pPD1	This study
PD134	∆*sphC* in PAO1	pPD23	This study
LAH304	∆*sphBCD* in PAO1	pPD54	This study
LAH301	∆*sphBCD* in PAO1	pPD55	This study
PD12	∆*sphBCD* in PAO1	pPD23	This study
AL51	*P.aeruginosa* PAO1 WT	pAL5	(PMID: 24465209) LaBauve *et al*.
MJ984	*P. aeruginosa* PA14 WT (new stock of MJ101)	–	(PMID: 7604262) Rahme *et al*.
PD49	∆*sphBCD* in PA14	–	This study
LAH 311	*P. fluorescens* WCS365 WT	–	This study
LAH 323	∆*sphBC* in WCS365	–	This study
LAH 313	*P. fluorescens* Pf-01 WT	–	This study
LAH 362	∆*sphBCD* in Pf-01	–	This study
LAH 314	*P. protegens* Pf-5 WT	–	This study
LAH 349	∆*sphBCD* in Pf-5	–	This study
JR124	*P. aeruginosa* PAO1 WT	pJM18	This study
JR125	*P. aeruginosa* PAO1 WT	pKSmScar6	This study
JR129	∆*sphBCD* in PAO1	pJM18	This study
JR131	∆*sphBCD* in PAO1	pKSmScar6	This study
JR268	∆*sphBCD* in PAO1	–	This study
MJ661	*S. aureus* WT	–	ATCC
PD207	*Caulobacter crescentus* WT NA1000	–	This study
PD209	∆*sphC* in *Caulobacter crescentus*	–	This study
PD113	*P. aeruginosa* PAO1 WT	pPD34	This study
PD108	∆*sphBCD* in PAO1	pPD34	This study
PD128	∆*sphBCD* in PAO1	pPD49	This study
PD117	∆*sphBCD* in PAO1	pPD35	This study

**Labplasmid ID**	**Description**		**Source**
pPD34	pUCP22, Pa replicative vector		(PMID: 1899844) Schweizer *et al*.
	pMQ30, allelic exchange		(PMID: 1899844) Schweizer *et al*.
pPD1	pMQ80, Pa replicative vector		(PMID: 16820502) Shanks *et al*.
pPD8	*sphBCD* in pMQ80, Pa replicative vector		This study
pPD23	*sphC* in pMQ80, Pa replicative vector		This study
pPD54	*sphBC* in pMQ80, Pa replicative vector		This study
pPD55	*sphCD* in pMQ80, Pa replicative vector		This study
pAL5	*sphA-lacZYA* reporter in pMQ80, Pa replicative vector		(PMID: 24465209) LaBauve *et al*.
pJM18	*sGFP2* in pUCP22		This study
pKSmScar6	*mScarlet-1* in pUCP22		This study
pPD49	Pa*sphBC* in pUCP22, Pa replicative vector		This study
pPD35	Cc*sphBC* in pUCP22, Pa replicative vector		This study

### General allelic exchange, chromosomal alterations and electroshock transformations

All allelic exchanges were completed using the pMQ30 non-replicative and counter-selectable vector [39]. Briefly, once constructs were cloned into the pMQ30 backbone, they were transformed into chemically competent S17 λ*pir Escherichia coli* by heat shock. For conjugation, donor and recipient cells were mixed, collected by centrifugation and resuspended in a small volume of LB and spotted onto LB plates to dry after which they were incubated overnight at 30 °C. Single crossover merodiploids were selected by plating on PIA with 50 µg ml^−1^ gentamicin at 37 °C, which also kills the donor *E. coli*. Two independent single crossovers for each allele were inoculated into LB and incubated at 37 °C for 3–4 h with shaking before plating on LB and LB with no NaCl and including 5% sucrose and incubated at 30 °C overnight. Sucrose-resistant colonies were then patched to LB with 5% sucrose and no NaCl plates (incubated at 30 °C) and LB with 50 µg ml^−1^ gentamicin plates (incubated at 37 °C) to identify and discard remaining merodiploids. Verification of strains from double crossovers was completed using PCR as described.

Allelic exchange vectors for deletion of *sphBCD* or *sphC* in PAO1 and PA14 were created by splice overlap extension (SOE) as we have described previously for other sphingosine-related genes [23]. Briefly, PCR fragments were amplified for both upstream and downstream of *sphBCD* or *sphC* and ligated into pMQ30 cut with either KpnI/HindIII or BamHI/HindIII. For *sphBCD* PCR fragment amplification, the upstream region was amplified with primers 2080 and 2083, while the downstream region was amplified using 2081 and 2083. For *sphC* PCR fragment amplification, the upstream region was amplified with primers 1022 and 1024, while the downstream region was amplified using 1023 and 1025. After verification by digest screening, plasmids were sequenced by Plasmidsaurus. Sequence-verified plasmids were transformed into chemically competent S17 λ*pir E. coli*, and allelic exchange was completed as described above, resulting in strains LAH 83.2 (PAO1 ∆*sphBCD*), PD49 (PAO1 ∆*sphBCD*) and PD47 (PAO1 ∆*sphC*).

Allelic exchange vectors for *sphBCD* deletion in *P. fluorescens* Pf-01 and *P. protegens* Pf-5 and *sphBC* deletion in *P. fluorescens* WCS365 were created using SOE as described above. After amplification and splice overlap, fragments were ligated into pMQ30 cut with KpnI/HindIII (for *P. fluorescens* WCS365 and *P. protegens* Pf-5) or XbaI/KpnI (for *P. fluorescens* PF-01). The *P. fluorescens* WSC365 *sphBC* upstream region was amplified with primers 2736 and 2737, while the downstream region was amplified using primers 2738 and 2739. The *P. fluorescens* Pf-01 *sphBCD* upstream region was amplified with primers 2740 and 2741, while the downstream region was amplified using 2742 and 2743. The *P. protegens* Pf-5 *sphBCD* upstream region was amplified using primers 2732 and 2733, while the downstream region was amplified using 2734 and 2735. After verification by digest screening, plasmids were sequenced by Plasmidsaurus. Sequence-verified plasmids were transformed into chemically competent S17 λ*pir E. coli*, and allelic exchange was completed as described above, resulting in strains LAH 323 (WSC365 ∆ *sphBC*), LAH 362 (Pf-01 ∆*sphBCD*) and LAH 349 (Pf-5 ∆*sphBCD*).

The *sphBCD*, *sphBC*, *sphCD* and *sphC* complementation constructs, pPD8, pPD54, pPD55 and pPD23, respectively, were generated by amplifying the appropriate region from genomic DNA using primer pairs 2726 and 2727, 2882 and 2883, 2726 and 2727 and 2511 and 2512, respectively, all cut with EcoRI and HindIII and independently ligated into similarly cut pMQ80. Plasmids with correct insert determined by PCR were sequenced (Plasmidsaurus), and correct plasmids were electrotransformed into target strains (Table 1). The vector control for all complementations was the empty pMQ80 vector.

The *sGFP2* construct, pJM18, and *mScarlet-1* construct, pKSmScar6, were built using HiFi (NEB) assembly using synthetic gene fragments (gBlocks) and ligated into pUCP22 digested with BamHI and EcoRI. pJM18 and pKSScar6 assemblies were verified by digest screening using HindIII and SacI, and digest-correct clones were sequenced (Plasmidsaurus) before electrotransformation into target strains (Table 1).

### Chemicals and notes on sphingolipid stability, solubility and handling

All media, media components and standard chemicals were purchased from Thermo Fisher or Sigma. The sphingoid bases sphingosine, phytosphingosine and sphinganine were purchased from Cayman Chemical and dissolved in 95% ethanol as aliquots of 50 mM stocks and stored at −20 °C. Storing as aliquots is important, as multiple freeze-thaw cycles lead to the loss of each of the sphingoid bases’ antimicrobial capacity and ability to stimulate gene induction via SphR [23]. Sphingoid bases were delivered to the culture vessel in ethanol, and then, the solvent evaporated to dryness, using air drying for multi-well plastic plates and a gentle stream of nitrogen gas for glass tubes.

### Determining IC_50_ for sphingosine, sphinganine and phytosphingosine in glass and plastic

*P. aeruginosa* strains were grown overnight at 37 °C shaking in MOPS media with 25 mM sodium pyruvate, 5 mM glucose and 20 µg ml^−1^ gentamicin. Cells from overnight cultures were collected via centrifugation, washed with MOPS media and resuspended in MOPS with 25 mM sodium pyruvate and 20 µg ml^−1^ gentamicin. Starting at an OD_600_ of 0.05, *P. aeruginosa* strains were grown for 18 h at 37 °C with horizontal shaking in either plastic 48-well plates or 13×100 mm glass tubes in the presence or absence of various concentrations of each sphingoid base. For the incubation periods, plates were covered with a sterile, breathable, adhesive microporous sealing film (USA Scientific) to allow for equal gas exchange for each well, while glass tubes were covered loosely with sterile aluminium foil. After 18-h incubations, OD_600_ was measured using a Synergy H1 or Synergy 2 (BioTek) plate readers. IC_50_ values were calculated in GraphPad Prism using the log(inhibitor) vs response – variable slope (four-parameter) curve fitting analysis.

### Kinetic growth assays

To measure growth kinetics, sphingoid bases were used at 200 µM. Prior to inoculation, *P. aeruginosa* strains were grown overnight at 37 °C, shaking in MOPS media with 25 mM sodium pyruvate, 5 mM glucose and 20 µg ml^−1^ gentamicin. Cells were collected via centrifugation, washed in MOPS media and resuspended in MOPS with 25 mM pyruvate and 20 µg ml^−1^ gentamicin, at a starting OD_600_ of 0.05 in 48-well plates sealed with breathable adhesive films. Absorbance for the film was corrected for by determining the difference between the absorbance post-film application to the read pre-application and subtracting that difference for each well and applying that to all reads for that well. Growth was measured via OD_600_ every 30 min with BioTek Synergy 2 or Synergy H1 plate readers set at 37 °C with orbital shaking before each read.

### Growth assays for other Pseudomonads, *Caulobacter* and heterologous complementation

To investigate the importance of *sphBCD* in other *Pseudomonas* strains and species, overnight cultures in MOPS media with 25 mM sodium pyruvate and 5 mM glucose were grown at 37 °C for *P. aeruginosa* strains and 30 °C for *P. protegens* and *P. fluorescens* strains. Cells were collected via centrifugation, washed in MOPS media and resuspended in MOPS media with 25 mM pyruvate (or 20 mM pyruvate, 10 mM glucose or 10 mM succinate when assessing catabolite repression). *Pseudomonas* strains and species were grown in sterile 13×100 mm borosilicate glass tubes or plastic 48-well plates for 18 h at 37 °C (for *P. aeruginosa* strains) or 30 °C (for *P. protegens* and *P. fluorescens* strains), with orbital shaking, in the presence or absence of sphingoid bases (200 µM final concentration) at a starting OD_600_ of 0.05. After 18-h incubations, growth was measured by OD_600_ using a Synergy H1 BioTek plate reader.

*Caulobacter crescentus* WT NA1000 and related ∆*sphC* were maintained at 30 °C on peptone-yeast extract plates containing 2 g l^−1^ Bacto Peptone, 1 g l^−1^ yeast extract, 1 mM MgSO_4_ and 0.5 mM CaCl_2_. Prior to assay set-up, strains were grown shaking at 30 °C overnight in M2 minimal salt medium [6.1 mM Na_2_HPO_4_, 3.9 mM KH_2_PO_4_, 9.3 mM NH_4_Cl, 0.5 mM MgSO_4_, 10 uM FeSO_4_ (EDTA chelate) and 0.5 mM CaCl_2_] with 0.2% glucose as the sole carbon source. To investigate the importance of *sphBC* in *C. crescentus*, overnight cultures in M2 minimal media were grown at 30 °C. Cells were collected via centrifugation and resuspended again in M2 minimal media at an OD_600_ of 0.05 and grown in sterile 13×100 mm borosilicate glass tubes at 30 °C with orbital shaking, in the presence or absence of sphingosine at varying concentrations. After 18-h incubations, growth was measured by OD_600_ using a Synergy H1 BioTek plate reader.

To investigate the ability of *sphBC* from *C. crescentus* to complement *P. aeruginosa* ∆*sphBCD* growth inhibition in the presence of sphingosine, overnight *P. aeruginosa* cultures in MOPS media with 25 mM sodium pyruvate, 5 mM glucose and 20 µg ml^−1^ gentamicin were grown shaking overnight at 37 °C. *sphBCD* complementation was assessed with native *P. aeruginosa* genes (PD128; PAO1 ∆*sphBCD* with Pa*sphBCD* on pUCP22) or *C. crescentus* homologues (PD117; PAO1 ∆*sphBCD* with Cc*sphBC* on pUCP22). Cells were collected via centrifugation, washed in MOPS media and resuspended in MOPS media with 25 mM pyruvate with 20 µg ml^−1^ gentamicin. *P. aeruginosa* strains were grown in sterile 13×100 mm borosilicate glass tubes for 18 h at 37 °C, with orbital shaking, in the presence or absence of sphingosine, at a starting OD_600_ of 0.05. After 18-h incubations, growth was measured by OD_600_ using a BioTek Synergy H1 plate reader.

### *sphA-lacZ* reporter assay

To determine the amount of sphingoid base remaining in culture when *sphBCD* is deleted, *sphA* transcriptional induction was measured using our previously described *sphA-lacZ* reporter assay and construct [23], and we refer to this as a sphingosine bioassay. *P. aeruginosa* was electrotransformed with the *sphA* promoter construct (pAL4) [23], and resultant colonies were grown overnight at 37 °C, shaking, in MOPS media with 25 mM sodium pyruvate, 5 mM glucose and 20 µg ml^−1^ gentamicin prior to induction. Cells were collected by centrifugation, washed in MOPS media and resuspended in MOPS media with 25 mM sodium pyruvate and 20 µg ml^−1^ gentamicin with or without lipid extracts from strains to be tested. Lipid extracts were collected for each strain after 18-h incubation in the presence or absence of sphingoid bases (200 µM final concentration). *β*-Galactosidase assays were then completed as previously described [40,41] using Miller’s method [42].

### TLC

To visualize the amount of sphingosine remaining in culture in the presence or absence of *sphBCD,* we used TLC. *P. aeruginosa* strains were grown overnight at 37 °C, shaking in MOPS media with 25 mM sodium pyruvate, 5 mM glucose and 20 µg ml^−1^ gentamicin. Cells were collected by centrifugation, washed in MOPS media and resuspended in MOPS media with 25 mM pyruvate and 20 µg ml^−1^ gentamicin at a starting OD_600_ of 0.05. Strains were grown for 18 h at 37 °C, with orbital shaking, in sterile foil-covered borosilicate 13×100 mm glass tubes with or without 200 µM sphingosine. After the incubation period, lipids were extracted from the whole cell culture using the Bligh and Dyer method [43]. Briefly, chloroform/methanol (1 : 2; v:v) was added, samples were vortexed and one volume of water was added. After briefly vortexing, samples were centrifuged for 10 min at 14 000 ***g***. After centrifugation, the lower organic fraction was collected and dried using N_2_ gas before final resuspension in 20 µl of ethanol. TLC silica gel 60 F_254_ plates (Sigma-Aldrich) were pre-run with acetone and dried, and lipid extracts were spotted onto the plate. After samples dried, plates were run in a closed glass chamber with chloroform/methanol/water (65 : 25 : 4; v:v:v) as the mobile phase. After the mobile phase approached the top of the plate, the plate was removed, dried and sprayed with ninhydrin solution (Acros Organics) to detect sphingosine by the reaction to its primary amine group.

### Liquid chromatography/electrospray ionization-tandem mass spectrometry (LC/ESI-MS/MS)

To directly quantify the levels of sphingosine remaining in culture in the presence and absence of *sphBCD*, LC/ESI-MS/MS was completed by Lipotype, Inc. (Germany). Strains were grown as per TLC, and, after incubation, samples were lysed at 4 °C for 10 min via bead beating with a vortex cell disruptor using 0.5 mm glass beads. Samples were stored at −80 °C until shipment to Lipotype, Inc. Before LC/ESI-MS/MS, samples were spiked with deuterated internal standards (including 0.25 ng sphingosine-d7). Methanol/isopropanol was added for protein precipitation, and the cleared solutions were analysed using an Agilent 1290 HPLC system with binary pump, multisampler and column thermostat with a Kinetex EVO C-18, 2.1×100 mm, 2.6 µm column using a gradient solvent system of ammonium carbonate (2 mM) and methanol. The flow rate was set at 0.4 ml min^−1^ and the injection volume was 1 µl. The HPLC was coupled with an Agilent 6495 Triplequad mass spectrophotometer (Agilent Technologies, Santa Clara, USA) with an electrospray ionization source. Analysis was performed with multiple reaction monitoring in positive mode, with at least two mass transitions for each compound. All sphingolipids were calibrated using individual standards. The Agilent MassHunter Quant software was used for quantification.

### *P. aeruginosa* competition assays

*P. aeruginosa* strains were grown overnight at 37 °C, shaking in MOPS media with 20 mM sodium pyruvate, 5 mM glucose and 20 µg ml^−1^ gentamicin prior to competition assay set-up. Cells were collected via centrifugation, washed three times in MOPS media and resuspended in MOPS media with 20 mM pyruvate and 20 µg ml^−1^ gentamicin and normalized to an OD_600_ of 0.5. Sterile borosilicate 13×100 mm glass tubes had vehicle or sphingosine, for a final concentration of 200 µM, dried as described above. To these tubes, 900 ml of MOPS media with 20 mM sodium pyruvate and 20 µg ml^−1^ gentamicin was added, followed by 50 µl each of 0.5 OD_600_ GFP and mScarlet-expressing *P. aeruginosa* for a total starting OD_600_ of 0.05. All cultures were grown at 37 °C, shaking for 18 h. At 0- and 18-h timepoints, OD_600_ and GFP (485/528 nm) and mScarlet (550/610 nm) fluorescent signals were measured using a Synergy H1 plate reader (BioTek). Background fluorescence for GFP and mScarlet was corrected by subtracting the signal from WT monoculture carrying the opposite fluorescent protein (mScarlet or GFP, respectively). Corrected fluorescence values were expressed as a percentage of monoculture of WT carrying GFP or mScarlet (set to 100%). Additionally, 20 µl aliquots of each culture were serially diluted in R2B and spot plated onto MOPS media agar plates with 20 mM sodium pyruvate and 5 mM glucose for c.f.u. counts at each timepoint. Total c.f.u. values were counted, and GFP-expressing colonies were detected by UV transillumination and appropriate excitation filter and imaged using a ChemiDoc XRS+Gel Imaging System (Bio-Rad). mScarlet-expressing colonies were calculated by subtracting GFP-expressing colonies from the total c.f.u. per millilitre.

### *P. aeruginosa*–*S. aureus* competition assays

*P. aeruginosa* was grown overnight in MOPS media with 20 mM sodium pyruvate and 5 mM glucose, shaking at 37 °C. Cells were collected by centrifugation, washed three times with MOPS media and added to 1 ml MOPS media with 20 mM pyruvate, 5 mM glucose and 20 µM sphingosine at a final OD_600_ of 0.05 to allow time for *sphBCD* induction. During this incubation, overnight 37 °C LB cultures of *S. aureus* were collected via centrifugation, washed three times with R2B and adjusted to an OD_600_ of 0.5 in R2B. *P. aeruginosa* was diluted into R2B±100 µM sphingosine for 1 h, shaking at 37 °C. After 1 h, *S. aureus* was added to an OD_600_ of 0.05 to the *P. aeruginosa*-containing media or R2B+/-100 µM sphingosine and grown for 5 h shaking at 37 °C. At 0 and 5 h of co-culture, 20 µl aliquots were serially diluted in R2B and spot plated onto both PIA and tryptic soy agar +7.5% NaCl to select for growth of *P. aeruginosa* and *S. aureus*, respectively, and c.f.u. was counted.

## Results

### The importance of *sphBCD* genes for *P. aeruginosa* growth in the presence of sphingosine and sphingosine analogues

**Fig. 2.**
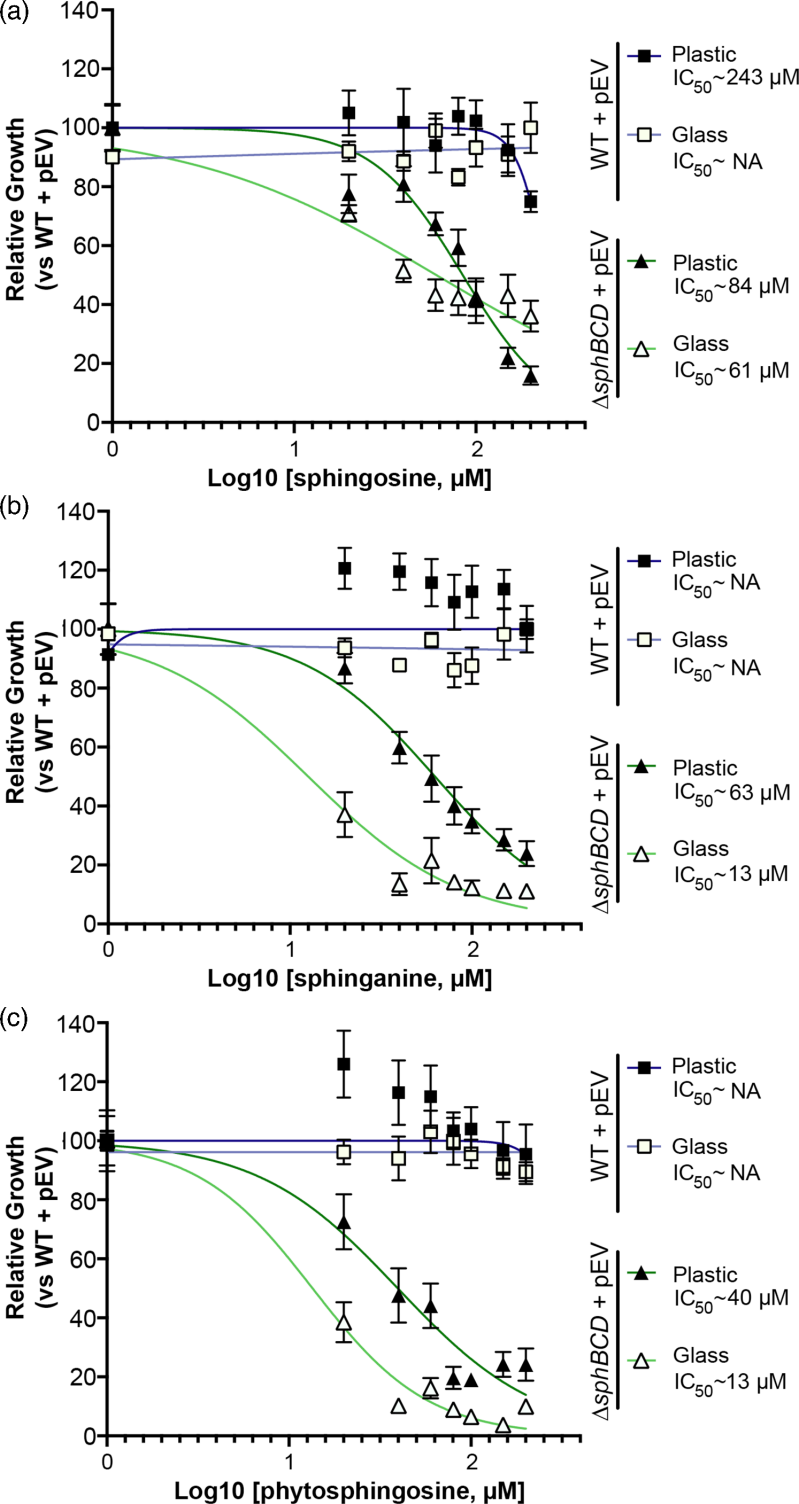
Concentration-dependent inhibition by sphingoid bases is dependent on the base and the culture vessel material. All panels show relative growth measured by OD_600_ as compared to the WT with empty vector (pEV) in the absence of sphingosine at the 18-h timepoint. The data shown here are for (**a**) sphingosine, (**b**) sphinganine and (**c**) phytosphingosine in either glass (open symbols) or plastic (closed symbols) in MOPS media with 20 mM pyruvate. The IC_50_ curve and estimated IC_50_s to the right of the plots are generated using variable-slope curve fitting in GraphPad Prism. If the calculated IC_50_ was above the solubility of sphingosine, it was listed as NA. Data points denote means summarizing three independent experiments, and error bars mark sd. pEV, empty vector pMQ80.

We previously reported the importance of *sphR* and *sphA* for resistance to sphingosine-dependent killing of *P. aeruginosa* PAO1 with a minor impact of *sphC* mutation [23]. Killing of *P. aeruginosa* by sphingosine requires specific media conditions that include a high divalent cation concentration and/or micellular sphingosine [17,23]. We observed that even in the absence of these very particular conditions in a modified MOPS minimal media and thus the absence of killing, sphingosine could strongly inhibit the growth of *P. aeruginosa* Δ*sphBCD* deletion mutants (Fig. 2a). The same protective role of *sphBCD* was observed during growth in the presence of sphinganine (Fig. 2b) and phytosphingosine (Fig. 2c) . The OD_600_ readings (non-normalized) for the data in Fig. 2 can be found in (Fig. S1, available in the online version of this article). In developing assays and testing various concentrations, we compared the growth in 13×100 mm glass tubes vs 48-well polystyrene multi-well plates, choosing this combination because the diameter of the two vessels is nearly identical and with the same sample volume, the surface area to volume ratios are very similar. When comparing these two conditions, we noted that inhibition by sphingosine was stronger when growth was conducted in glass rather than in plastic at a given concentration of sphingosine. We note this observation to provide context for data from assays conducted in one or the other condition.

**Fig. 3.**
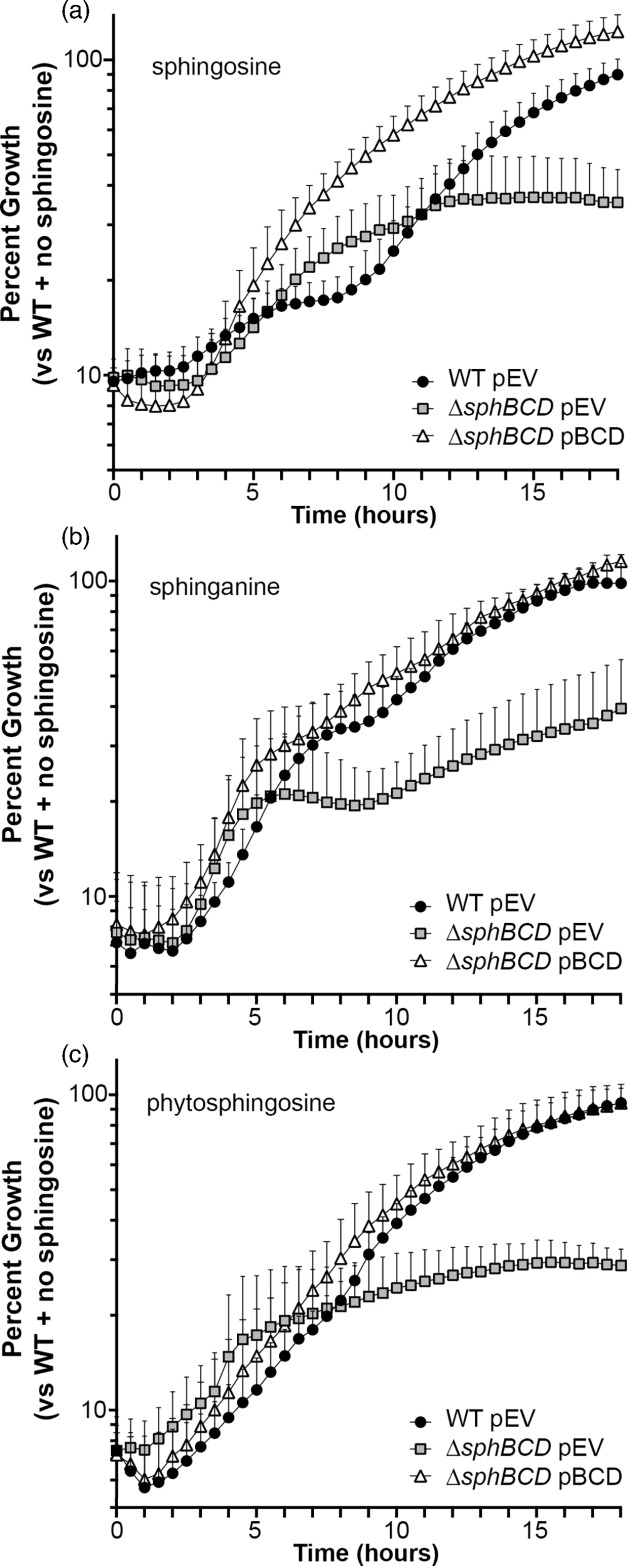
Kinetic growth assessment of WT, mutant and complemented strains in the presence of sphingoid bases. All panels are 18-h timecourses measuring the relative growth of each strain at each timepoint as measured by OD_600_ compared to the WT with empty vector (pEV) in the absence of sphingosine at the 18-h timepoint. The data shown here are for 200 µM (**a**) sphingosine, (**b**) sphinganine or (**c**) phytosphingosine in MOPS media with 20 mM pyruvate. Growth curves in MOPS pyruvate in the absence of sphingoid bases are presented in Fig. S3. Data points denote means summarizing three independent experiments, and error bars mark sd with only the bars above the mean shown for figure clarity. pEV, empty vector pMQ80; pBCD, vector-containing *sphBCD*.

Deletion of the *sphBCD* operon increased susceptibility to sphingosine and close analogues when measured at 18-h post-inoculation Fig. 2, and we wanted to examine the kinetics of this growth inhibition by measuring growth over time. We measured growth with 100% set as WT OD_600_ in the absence of sphingosine at 18 h. At 200 µM sphingosine, the *sphBCD* deletion mutant shows initial growth that starts to plateau after about 10 h, while WT has a delay in growth before resumption of a nearly normal growth rate. The complementation strain has no substantial delay (Fig. 3a). The *sphBCD* deletion is also defective for growth in the presence of sphinganine (Fig. 3b) and phytosphingosine (Fig. 3c). The OD_600_ readings (non-normalized) for the data in Fig. 3 can be found in Fig. S2. Neither sphinganine nor phytosphingosine shows the strong delay in WT growth as with sphingosine, and, while ∆*sphBCD* growth in phytosphingosine shows the same plateau as for sphingosine (compare Fig. 3c with Fig. 3a), the ∆*sphBCD* strain can grow slowly in the presence of sphinganine with a substantial delay. A direct comparison of the growth of each of these three strains in the absence of sphingosine is presented in Fig. S3, showing no inherent growth defect.

### The critical role of *sphC* for growth in the presence of sphingosine

**Fig. 4.**
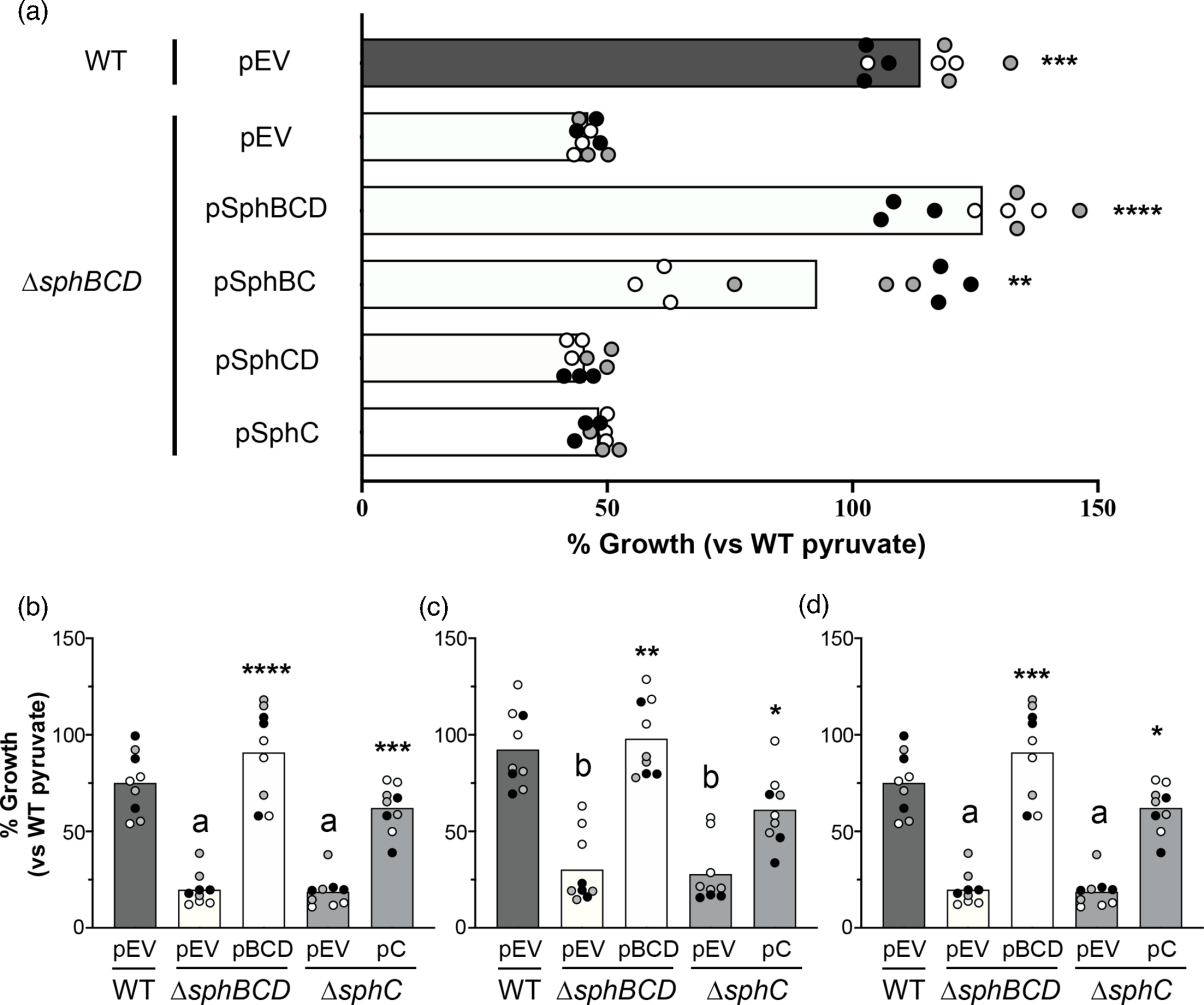
The *sphBC* genes are critical for WT levels of growth in the presence of 200 µM sphingoid bases. (**a**) Complementation analysis of ∆*sphBCD* shows significant complementation only with plasmids expressing *sphB* and *sphC*, while *sphD* appears dispensable for growth at 18 h normalized to WT empty vector growth in MOPS pyruvate set as 100%. (**b–d**) Deletion of *sphC* phenocopies deletion of *sphBCD* and can be complemented by *sphC* on a plasmid. This phenotype is shared between the sphingoid bases (**b**) sphingosine, (**c**) sphinganine and (**d**) phytosphingosine. Eighteen-hour growth was normalized to WT empty vector growth in MOPS pyruvate set as 100%. For all panels, all data points are shown and are coloured by experiment with white circles for all replicates from experiment #1, grey from experiment #2 and black from experiment #3. Only the means for each experiment are used in the statistical analyses for these panels (*n*=3 per condition). For (**a**), significance was noted as **P<0.01, ***P<0.001 and ****P<0.0001 calculated from ANOVA with Dunnett’s post-test with ∆*sphBCD* pEV as the comparator. For (**b)–(d**), significance was noted as **P<0.01, ***P<0.001 and ****P<0.0001 for comparisons of each complemented strain to its empty vector control, while significant difference to WT with empty vector was noted as a, *P*<0.0001, and b, *P*<0.01. Analysis of (b)–(d) was conducted using ANOVA and Tukey’s post-test comparing all groups. pEV, empty vector pMQ80; pBCD, vector-containing *sphBCD;* pC, vector-containing *sphC*.

Deletion of *sphBCD* can be complemented by plasmids carrying *sphBCD* or a plasmid carrying *sphBC*, but not other single genes from the locus (Fig. 4a), supporting *sphB* and *sphC* as required components for resistance to sphingosine. Deletion of *sphC* alone phenocopies ∆*sphBCD* and *sphC* complements this phenotype in ∆*sphC* (Fig. 4b). Similar to sphingosine, deletion of *sphC* results in growth inhibition by the sphingosine analogues sphinganine and phytosphingosine, and these phenotypes can be complemented (Fig. 4c, d). The OD_600_ readings (non-normalized) for the data in can be found in Fig. S4. These data support the role of *sphBC* in resistance to growth inhibition by antimicrobial sphingoid bases.

### *sphBC* are important for the metabolism of sphingosine to a non-toxic metabolite

**Fig. 5.**
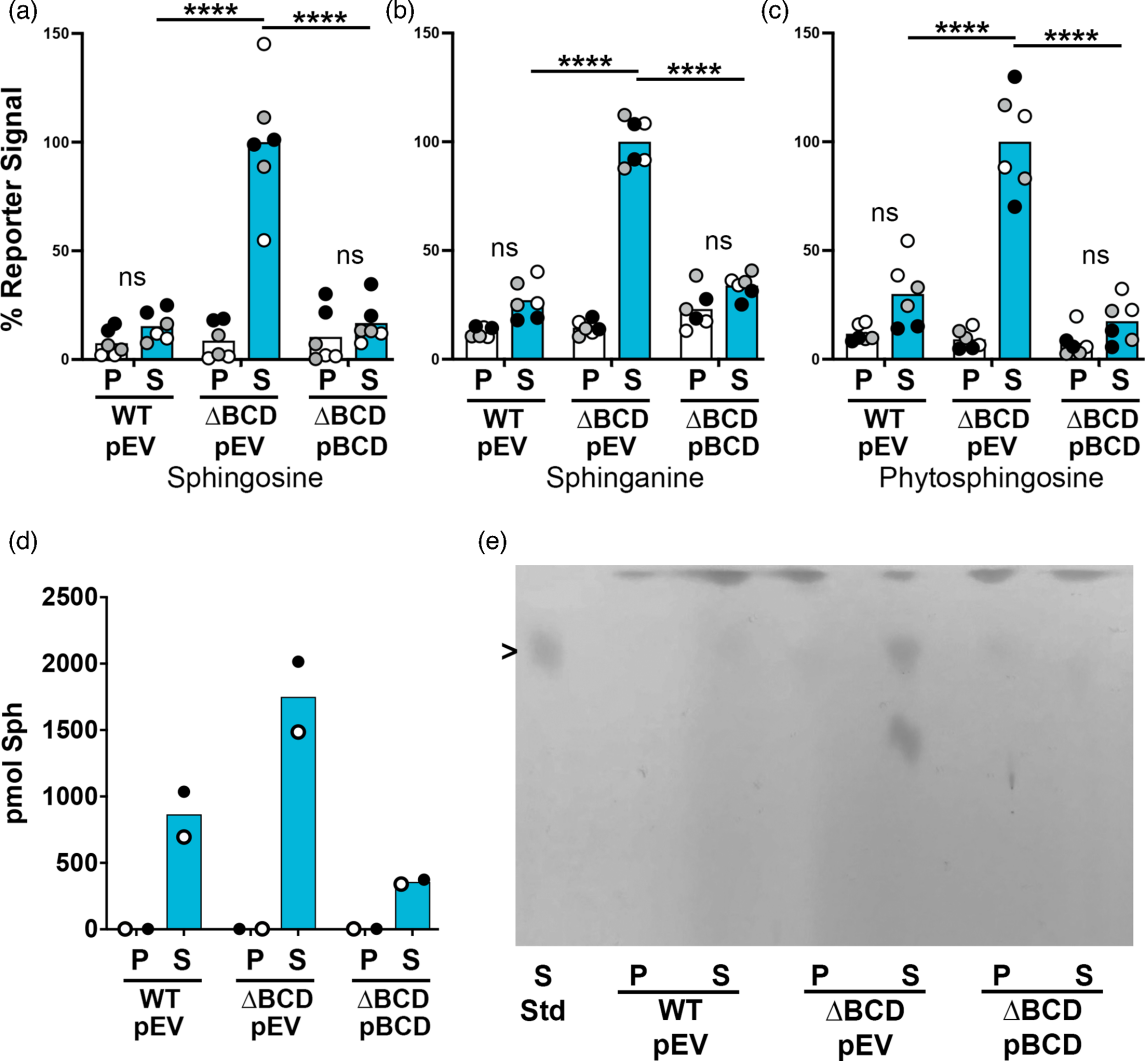
Metabolism of sphingoid bases is dependent on the presence of *sphBCD*. (**a–c**) Determination of sphingoid bases remaining in the cultures in MOPS media with 20 mM pyruvate after 18 h of incubation as measured using the *sphA-lacZ* reporter assay, (**d**) liquid chromatography-mass spectrometry (LC-MS) and (**e**) TLC with the migration of the sphingosine standard noted with the arrowhead to the left of the image. Statistical significance was noted as ****P<0.0001 using a two-way ANOVA with Tukey’s post-test comparing all groups. For panels (a)–(d), all data points are shown and are coloured by experiment with white circles for all replicates from experiment #1, grey from experiment #2 and black from experiment #3. Only the means for each experiment are used in the statistical analyses for these panels (*n*=3 per condition, except the LC-MS, for which only two replicates were run and are therefore not statistically analysed). The spot that runs below sphingosine in the ∆*sphBCD* mutant TLC lane (in e) is an unknown amine-containing lipid and did not run similarly to any of our sphingolipid standards. ns, not significant; P, pyruvate (control); S, sphingosine; ∆BCD, ∆*sphBCD*; pEV, empty vector pMQ80; pBCD, vector-containing *sphBCD*.

While there were many potential mechanisms by which *sphBC* could provide sphingosine resistance, one potential mechanism was the metabolism of sphingoid bases to a compound that was not growth inhibitory. The *sphC* gene encodes a twin-arganine translocon (TAT)-secreted periplasmic oxidoreductase [44], and *sphB* encodes a Sec-secreted periplasmic cytochrome c5-like protein, predicted to be a lipoprotein. These predicted functions suggested a role for oxidation of some compound in the periplasm, potentially sphingosine or a compound required for subsequent sphingosine metabolism. Sphingosine is depleted from supernatants and cell culture extracts of WT cells (Fig. 5 and also observed in [45]), while sphingosine and close analogues accumulate in cell culture extracts of ∆*sphBCD*, as measured by bioassay (Fig. 5a, c). The SphR bioassay is based on our previous work that an SphR-controlled reporter gene responds to sphingosine generating a reporter signal proportional to the concentration of sphingosine (Fig. 1E from [23]). The bioassay measures are supported by liquid chromatography-mass spectrometry (Fig. 5d) and TLC (Fig. 5c). These data support the role of *sphBC* in sphingosine metabolism to a non-toxic product, as functional *sphBC* (WT) results in no substantial growth inhibition and absence of the added sphingosine.

### Phylogenetic distribution of the *sphBCD* genes and their roles in other species

**Fig. 6.**
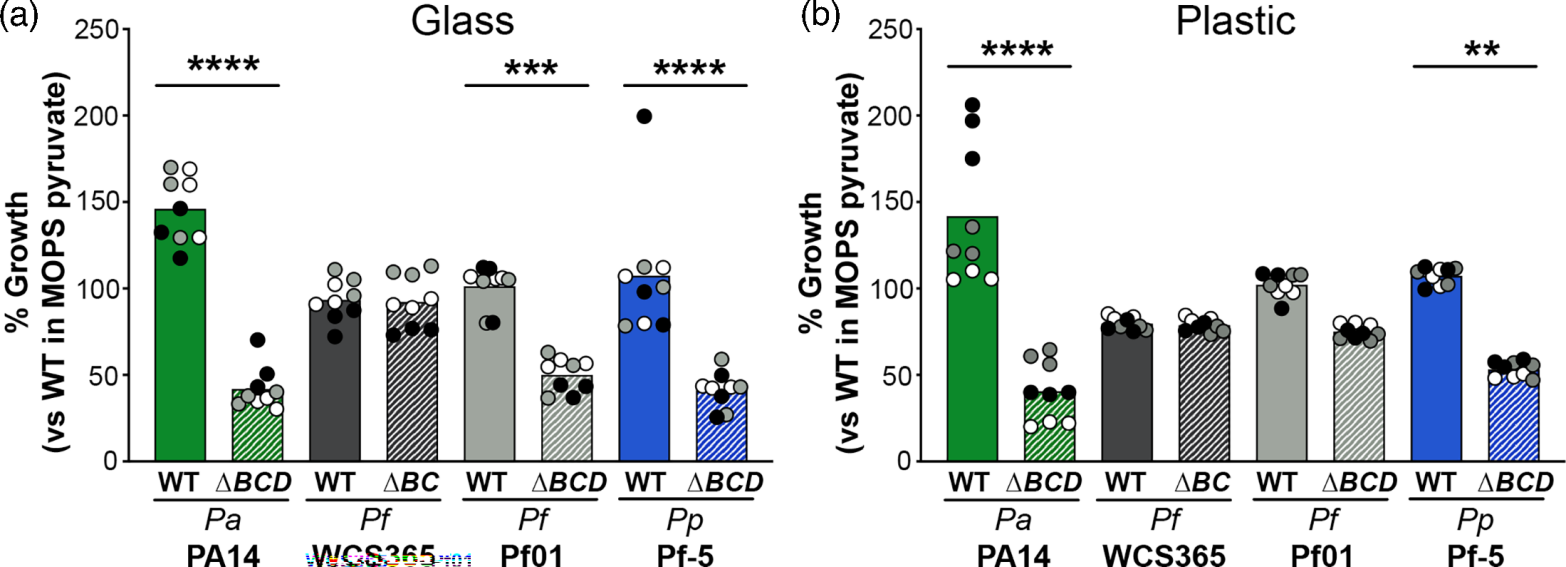
The role of *sphBC* in other Pseudomonads. The growth WT and mutant for each strain in 200 µM sphingosine in MOPS media with 20 mM pyruvate are shown normalized to that strain’s growth in MOPS pyruvate media. As seen for *P. aeruginosa* PAO1 (Fig. 2), the culture vessel material impacts the potency of sphingosine for some strains. Significance was noted as **P<0.01, ***P<0.001 and ****P<0.0001 calculated from ANOVA with Sidak’s post-test comparing WT to mutant within each strain. For both panels, all data points are shown and are coloured by experiment with white circles for all replicates from experiment #1, grey from experiment #2 and black from experiment #3. Only the means for each experiment are used in the statistical analyses for these panels (*n*=3 per condition). ∆BCD, ∆*sphBCD*; ∆BC, ∆*sphBC*; Pa, *P. aeruginosa*; Pf, *P. fluorescens*; Pp, *P. protegens*.

The *sphBCD* genes are present in all sequenced *P. aeruginosa* and are also present in most non-*aeruginosa* Pseudomonads using the *Pseudomonas* genome browser [46]. As *sphB* and *sphC* encode proteins in large families, true orthology is difficult to assess, particularly in the absence of any direct understanding of substrate interaction in the case of SphC. Co-occurrence searches with STRING [47] yield quite a large number of hits in the *Firmicutes*; *Actinobacteria*; and Alpha-, Beta- and Gamma-*Proteobacteria*, but nothing outside of those groups. Manual searching through the resultant genes suggested that some could be orthologues, including a putative SphC of *C. crescentus*, described below, while others are likely unrelated to sphingosine. Therefore, we first focused on assessing the function of the *sphBCD* genes in other Pseudomonads, including *P. fluorescens* WCS365, which does not have an *sphD* orthologue in its *sphBC* operon. Deletion of the *sphBCD* genes from *P. aeruginosa* PA14 and *P. protegens* Pf-5 showed a growth defect in the presence of 200 µM sphingosine regardless of culture vessel material (Fig. 6a, b). The *sphBCD* deletion mutant of *P. fluorescens* Pf01 showed less growth than WT in the presence of sphingosine in each vessel material, but the difference was only significant in glass (Fig. 6a). Deletion of *sphBC* in *P. fluorescens* WCS365 did not show a phenotype. The OD_600_ data from these experiments are shown in Fig. S5. These data support the role of *sphBC* in resistance to sphingosine beyond *P. aeruginosa*, but the presence of these genes does not necessarily predict their importance for growth in the presence of sphingosine. We deleted the *sphC* gene from *C. crescentus,* but the measured effect was significant only within a very small sphingosine concentration range (Fig. S6A). We also tested the heterologous expression of *C. crescentus sphBC* to attempt complementation of *P. aeruginosa ∆sphBCD*. For *C. crescentus* putative *sphBC*, the native Sec- and TAT-signal sequences encoded in *sphB* and *sphC*, respectively, were swapped for the Sec- and TAT-signal sequences from *P. aeruginosa sphB* and *sphC*. While data show a trend towards partial rescue, the impact of *C. crescentus sphBC* in *P. aeruginosa* was not statistically significant (Fig. S6B).

### Detoxification of sphingosine is a public good

**Fig. 7.**
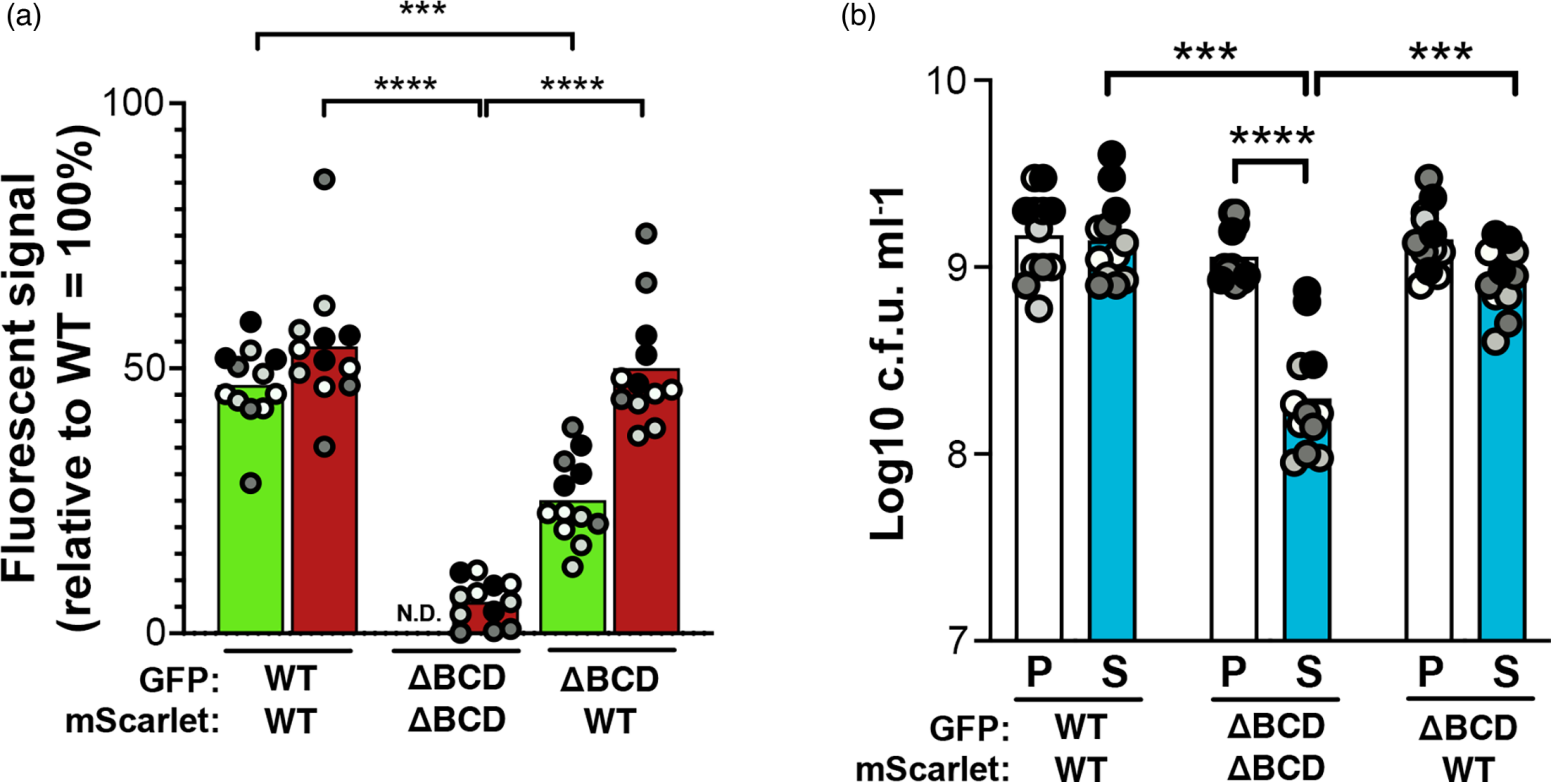
WT sphingosine detoxification can protect co-cultured ∆*sphBCD* from growth inhibition by sphingosine. (**a**) Fluorescence signal for GFP and mScarlet normalized to a monoculture of WT carrying GFP or mScarlet, respectively. GFP signal is shown with light green bars, and mScarlet signal is shown with dark red bars. The strain carrying each fluorescent protein is labelled below the graph. (**b**) c.f.u. counts of GFP-expressing colonies in the presence (S) or absence (P) of sphingosine. The strain carrying each fluorescent protein is labelled below the graph. Significance was noted as ***P<0.001 and ****P<0.0001 calculated from ANOVA with Tukey’s post-test comparing within and between co-culture groups. For both panels, all data points are shown and are coloured by experiment with white circles for all replicates from experiment #1, light grey from experiment #2, dark grey from experiment #3 and black from experiment #4. Only the means for each experiment are used in the statistical analyses for these panels (*n*=4 per condition). ∆BCD, ∆*sphBCD*; P, pyruvate (control); S, sphingosine; N.D., not detectable.

**Fig. 8.**
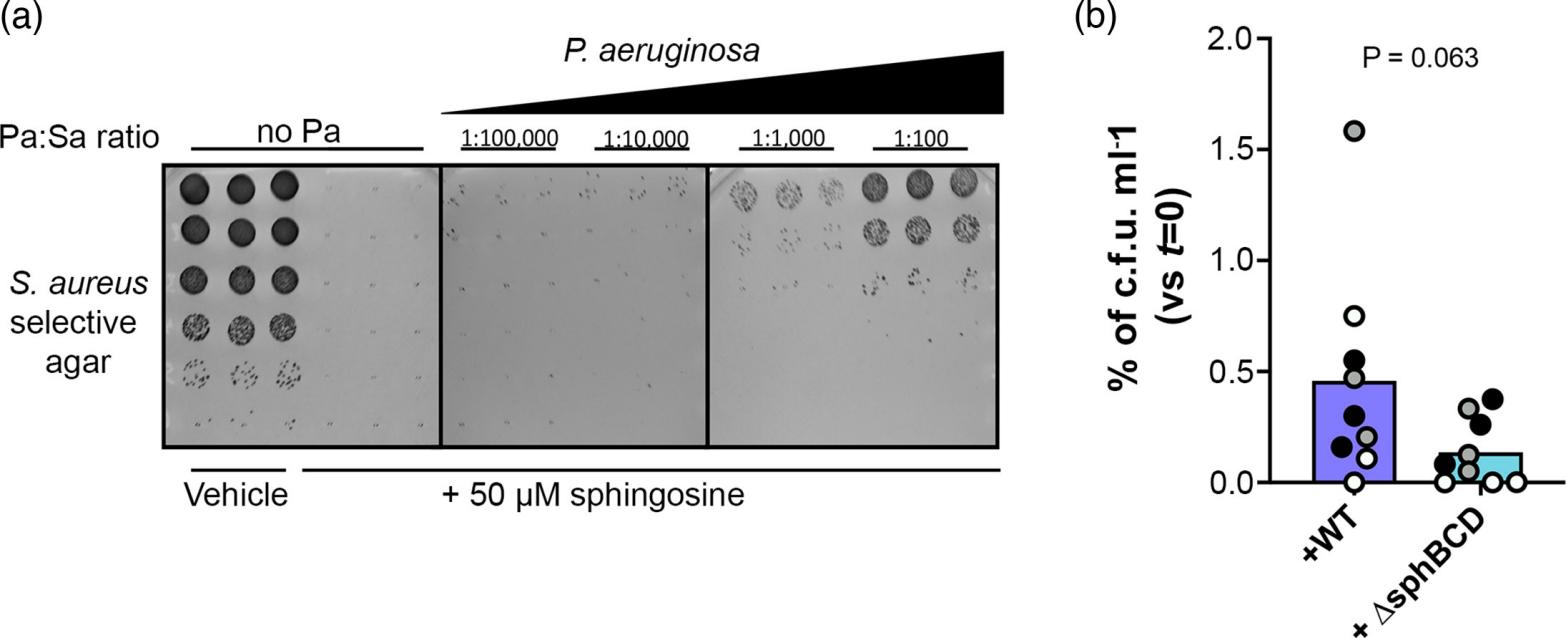
*P. aeruginosa* can protect *S. aureus* from complete killing by sphingosine. (**a**) Titration of WT *P. aeruginosa* into the sphingosine-containing media for 1 h prior to *S. aureus* inoculation protected a small proportion of *S. aureus* from the lethal effects of 5 h in the presence of 50 µM sphingosine in a *P. aeruginosa* inoculum-dependent manner. (**b**) The proportion of the initial *S. aureus* population protected by *P. aeruginosa* WT trended higher than the proportion protected by ∆*sphBCD*. All data points are shown and are coloured by experiment with white circles for all replicates from experiment #1, grey from experiment #2 and black from experiment #3. Only the means for each experiment were used for a t-test to statistically analyse these data (*n*=3 per condition), and thus, the technical replicates with no c.f.u. are averaged to a non-zero number for each experiment (even for experiment #1, where only one replicate had countable colonies).

The *sphBC* genes have a role in the metabolism of sphingosine to a product that is not growth inhibitory, which suggests that cells capable of sphingosine metabolism could potentially protect cells that cannot otherwise metabolize sphingosine from sphingosine’s antimicrobial effects. WT *P. aeruginosa* partially protects ∆*sphBCD* from sphingosine growth inhibition as measured by both fluorescent signal (Fig. 7a) and c.f.u. (Fig. 7b), and the same effect was seen when the fluorescent markers were swapped between the strains (Fig. S7). *P. aeruginosa* could likewise protect the sphingosine-susceptible *S. aureus* (Fig. 8a). While protection of *S. aureus* by ∆*sphBCD* trended lower than WT (Fig. 8b), this did not reach statistical significance given the assay variability. We specifically chose the timing of our co-culture (5 h, with details in the Methods) to precede the often-observed killing of *S. aureus* by *P. aeruginosa*, but this antagonistic interaction certainly is an issue that confounds even these short timepoints. Additionally, under these conditions, we do not know the sphingosine concentration remaining to which *S. aureus* is subjected.

## Discussion

Sphingoid bases, including sphingosine, are important antimicrobial compounds on epithelial surfaces of mammals [1,12] and are also produced by plants and released into the rhizosphere [48]. Here, we report the identification of the *P. aeruginosa sphBCD* operon as necessary for metabolism, and thus detoxification, of sphingosine and other sphingoid bases, showing that functional *sphBCD* is needed for WT levels of growth in the presence of sphingoid bases. These conclusions are supported by growth studies, complementation and measurements of sphingosine metabolism. WT *P. aeruginosa* can also protect susceptible bacteria from sphingosine, pointing to a potential role in mixed microbial communities.

The work presented here focuses on conditions wherein sphingosine inhibits growth but is not bactericidal for either WT or ∆*sphBCD*. These conditions are quite different than those required for *P. aeruginosa* killing by sphingosine by us and others, which typically use very high divalent cation concentrations and are dependent on the phase of the lipid [17,23]. In this current work, sphingosine is dried onto the vessel surface, allowing vehicle evaporation prior to adding media and *P. aeruginosa,* which results in growth inhibition rather than killing, though others have also noted bacterial growth inhibition rather than killing for sphingoid bases [48]. Therefore, while the concentration of sphingosine in the entire well is listed in our experiments, the concentration of free sphingosine in the liquid phase at any given point in time is unknown. Our model may not mimic the antimicrobial activity of sphingosine in liquid-covered epithelium, like in the lung [12], and might be a closer mimic to the antimicrobial activity of sphingosine on the skin with a temporary covering of sweat [3]. In a similar manner, our model is likely closer to the behaviour of plant-derived sphingoid bases in non-saturated soils. The importance and properties of the vessel material underline the difference of our model, where there are noticeable differences in concentration-dependent inhibition and growth phenotype depending on whether the culture vessel was glass or plastic. Because of the very different conditions in our model, the phenotypes shown here are not directly comparable to the killing phenotypes we previously reported [23] or to the *P. aeruginosa* killing presented by others [17]. In our previous work, an *sphC* mutant had a small but measurable defect in the sphingosine killing assay, while ∆*sphR* and ∆*sphA* mutants were very susceptible to sphingosine killing. However, in the growth inhibition assay, the *sphA* mutant has no phenotype (Fig. S8A). Additionally and interestingly, while *sphBCD* can be induced by sphingosine in an *sphR*-dependent manner [23], *sphR* is not required for growth in the presence of sphingosine (Fig. S8B), suggesting either that basal transcription is sufficient for growth or that there is another regulator inducing *sphBCD*, perhaps related to the envelope stress response. We think that these differences in phenotypes for sphingosine-related mutants in the two sphingosine response models, killing vs growth inhibition, are likely biologically important and may reflect the management of sphingosine under different environmental conditions. We also note that the carbon source in minimal media impacts the effect of sphingosine on PAO1 growth with less impact of sphingosine when grown using a carbon source, which enables faster growth (Fig. S9), though complementation with *sphBCD* still improves growth even when the level of inhibition is small (i.e. in MOPS+succinate). This effect of carbon source could be due to either a faster growth rate or more rapid accumulation of cell mass that could dilute the effectiveness of sphingosine – these are conjectures and would need to be formally tested.

Our genetic analysis implicates SphC and SphB as the critical proteins for sphingosine resistance encoded in the *sphBCD* operon, as deletion of *sphC* phenocopies ∆*sphBCD* (Fig. 4b–d) and only vectors containing both *sphC* and *sphB* can complement ∆*sphBCD* (Fig. 4a). SphC is predicted to be an FMN-linked oxidoreductase and is known to be TAT secreted and localized to the periplasm [44]. SphB is a predicted lipoprotein cytochrome c with a Sec signal sequence. Based on the data presented here and the presence of the *sphBCD* operon in the sphingosine:SphR regulon [23], we predict that SphC can oxidize sphingosine to a metabolite that is non-toxic and the electron needed for this oxidation is replenished by SphB. Some evidence supporting that SphC and SphB might be partners is that while plasmid-borne *sphC* can complement ∆*sphC*, it is not as strong a complementation as plasmid-borne *sphBC* complementation of ∆*sphBCD* (Fig. 4). This could be explained by a stoichiometry mismatch between SphC and SphB. As to why the plasmid carrying *sphBC* does not complement as well as the plasmid carrying *sphBCD*, we are not sure, though since we have not measured transcript and protein levels generated from these constructs, it could simply be a difference in functional expression. It is interesting to note that the two organisms that we tested that carry only *sphBC* in an operon, *P. fluorescens* WCS365 and *C. crescentus*, compared to those with *sphBCD*, show little to no effect of the *sphBC* deletion on growth in the presence of sphingosine (Figs 6 and S6).

Multiple attempts to identify the direct metabolite of sphingosine were unsuccessful, perhaps because one potential product would be a very reactive aldehyde aldol. While our data here underscore the necessity of *sphBC* for sphingosine metabolism and normal levels of *P. aeruginosa* growth in the presence of sphingosine, we currently have no evidence that *sphBC* are sufficient for sphingosine metabolism. This leaves open the possibility that SphB and SphC act indirectly to detoxify sphingosine. Complementation of *P. aeruginosa* ∆*sphBCD* with secretion-adapted *sphBC* homologues from *C. crescentus* showed no significant effect (Fig. S6). There are many reasons this heterologous complementation might have failed yet be non-informative, including poor protein folding in the heterologous host, secretion failure despite the attempt at secretion adaptation of each sequence to the heterologous host, rapid degradation of one or both proteins or, in the case of SphB, failure to interact with the unknown inner membrane electron donor in the heterologous host. Additionally, while the *C. crescentus* putative SphB and SphC are homologous to *P. aeruginosa* SphB (44% identity, 55% positive) and SphC (42% identity, 58% positive), it is unknown whether they are orthologous.

When we examined *P. aeruginosa* PA14 and other *Pseudomonas* species, we noted that while *sphBCD* deletion led to poorer growth in the presence of sphingosine for *P. aeruginosa* PA14, *P. fluorescens* Pf01 and *P. protegens* Pf-5, deletion of *sphBC* in *P. fluorescens* WCS365 had no phenotype (Fig. 6). Additionally, the magnitude of the phenotype differed between species and, like for *P. aeruginosa*, was dependent on the culture vessel material. Combining these findings with the observation that *P. aeruginosa* ∆*sphBCD* can still grow in the presence of sphingosine, albeit not to the same extent as WT, we conclude that there are other proteins or cellular processes that can function independently of *sphBCD* to abrogate the effects of sphingosine. As an example, in *P. fluorescens* WCS365, there is no *sphD* homologue at the locus and there is a very minimal decrease in the growth of either WT or ∆*sphBC* in the presence of sphingosine. This strain must have an alternate mechanism to resist growth inhibition by sphingosine.

Regardless of whether SphC and SphB directly act on sphingosine, *sphBC*-dependent sphingosine metabolism depletes sphingosine from the media (Fig. 5). Such sphingosine depletion led us to hypothesize that the metabolism of sphingosine by WT cells would protect sphingosine-susceptible bacteria in co-culture, which we observed in a co-culture of WT and ∆*sphBCD* cells (Fig. 7). Similarly, *S. aureus* is completely killed by 50 µM sphingosine under the conditions of our assay, but a small percentage can be protected by *P. aeruginosa*. While fewer *S. aureus* are protected by ∆*sphBCD*, variation in the means makes the contribution of *sphBCD* to this protection not statistically significant. One of the caveats of this *P. aeruginosa–S. aureus* co-culture is that, for these lab isolates, *P. aeruginosa* eventually kills the *S. aureus* [49,51]. Future work will look at co-isolates of these species from the same patient samples, where apparently peaceful co-existence is common [52]. Since many bacteria and some fungi are susceptible to sphingoid bases [3,48], sphingoid base detoxification in areas of very high sphingosine concentration (skin and rhizoplane) might contribute to community structure and composition.

Our identification and characterization of the sphingoid base-dependent phenotype of *sphBCD* and *sphC* mutants is an important step in our understanding of bacterial manipulation of sphingolipids. However, there remain a number of important and unaddressed issues identified during our work, including the biochemical mechanism behind SphC and SphB function, the identity and role of *sphBC*-independent sphingosine management systems in *P. aeruginosa* and other Pseudomonads and the contributions of sphingosine detoxification to spatial architecture in sessile communities.

## supplementary material

10.1099/mic.0.001520 Uncited Supplementary Material 1.

## Abbreviations

CF: cystic fibrosis
LB: lysogeny broth
PIA: *Pseudomonas* isolation agar
SOE: splice overlap extension
TAT: twin-arganine translocon
