# Supplementary material for: The Pseudomonas aeruginosa sphBC genes are important for growth in the presence of sphingosine by promoting sphingosine metabolism
Source: Microbiology (Reading). 2025 Jan 10;171(1):001520. doi: 10.1099/mic.0.001520 (PMC11893366; doi:10.1099/mic.0.001520)
Supplement: Uncited Supplementary Material 1. [file mic-171-01520-s001.pdf]

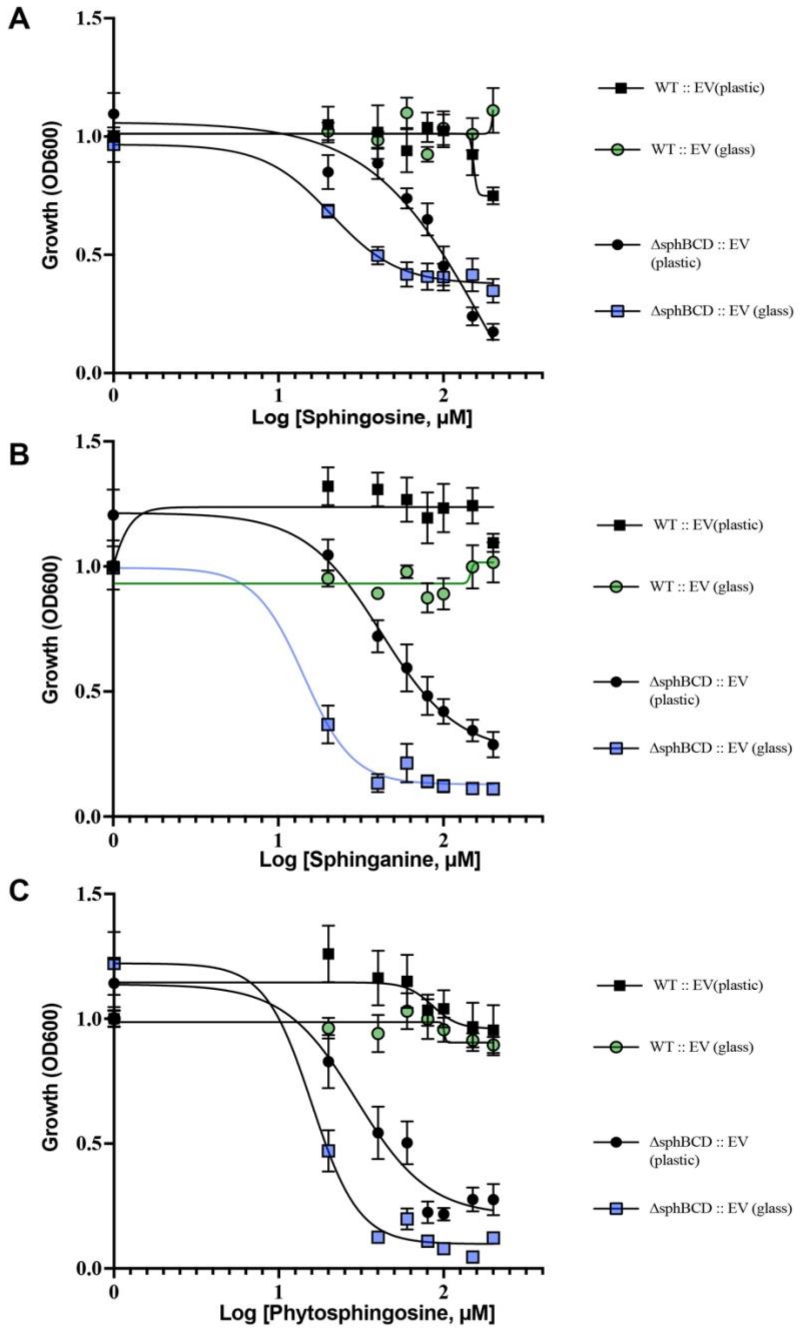

Supplemental Figure S1: Concentration-dependent inhibition by sphingoid bases is dependent on the base and the culture vessel material. All panels show OD_{600}. The data shown here are for **(A)** sphingosine, **(B)** sphinganine, and **(C)** phytosphingosine in either glass (open symbols) or plastic (closed symbols) in MOPS media with 20 mM pyruvate. The curves were generated using variable-slope curve fitting in GraphPad Prism. Data points denote means summarizing three independent experiments and error bars mark standard deviation. Abbreviations: pEV, empty vector pMQ80.

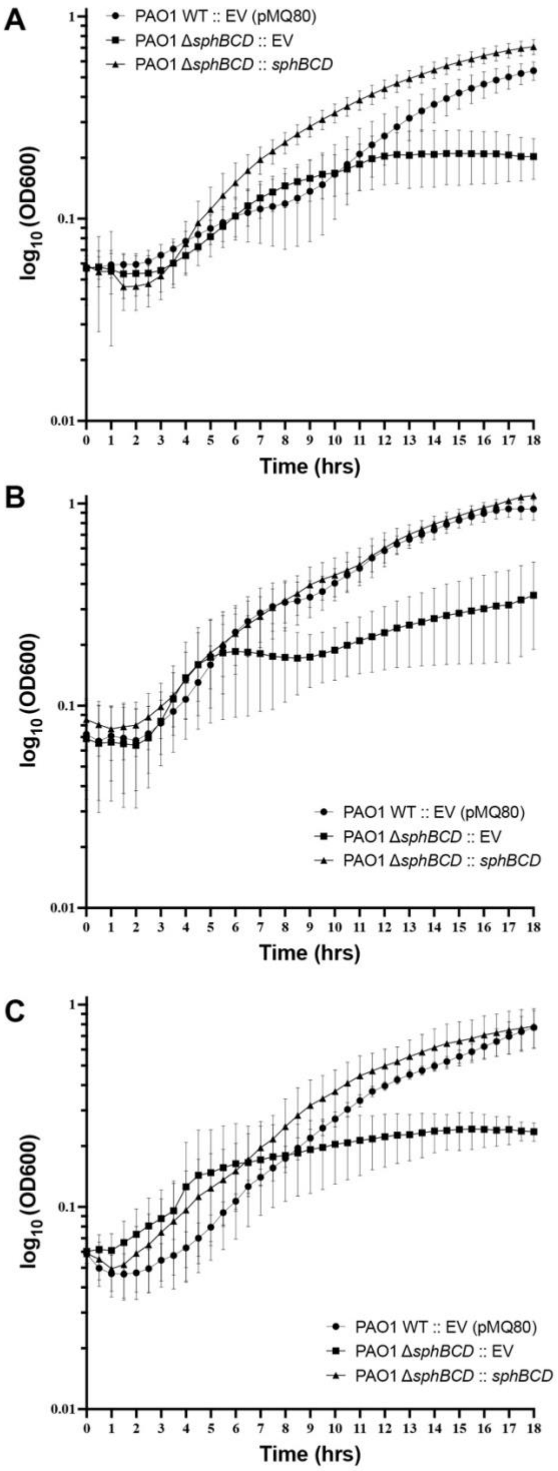

Supplemental Figure S2: Kinetic growth assessment of wild-type, mutant, and complemented strains in the presence of sphingoid bases. All panels are 18-hour timecourses measuring OD_{600}. The data shown here are for 200 µM of **(A)** sphingosine, **(B)** sphinganine, or **(C)** phytosphingosine in MOPS media with 20 mM pyruvate. Data points denote means summarizing three independent experiments and error bars mark standard deviation with only the bars above the mean shown for figure clarity. Abbreviations: pEV, empty vector pMQ80; pBCD, vector containing *sphBCD*.

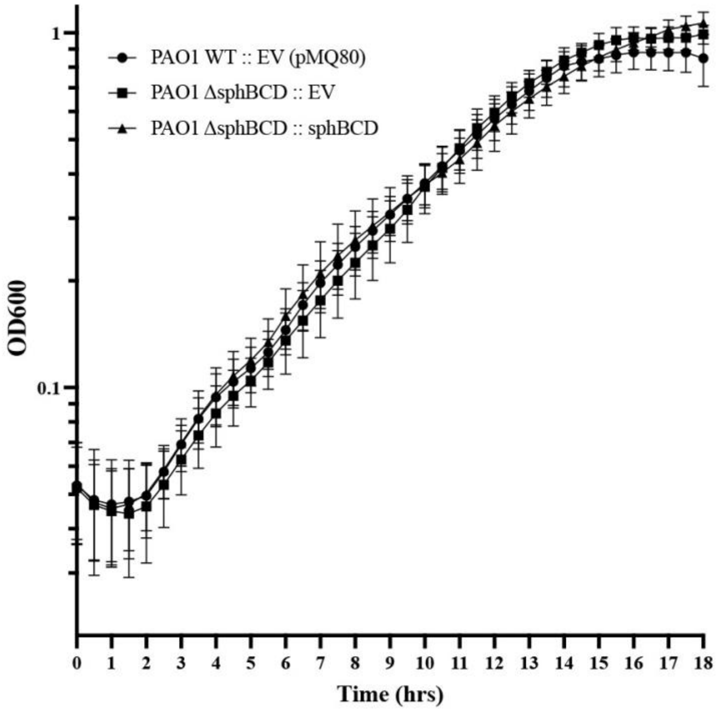

Supplemental Figure S3: Kinetic growth assessment wild-type, mutant, and complemented strains in the absence of sphingoid bases. Panels shows 18-hour growth timecourse in MOPS pyruvate media measuring growth of each strain by OD_{600}. Abbreviations: EV, empty vector pMQ80; sphBCD, vector containing *sphBCD*.

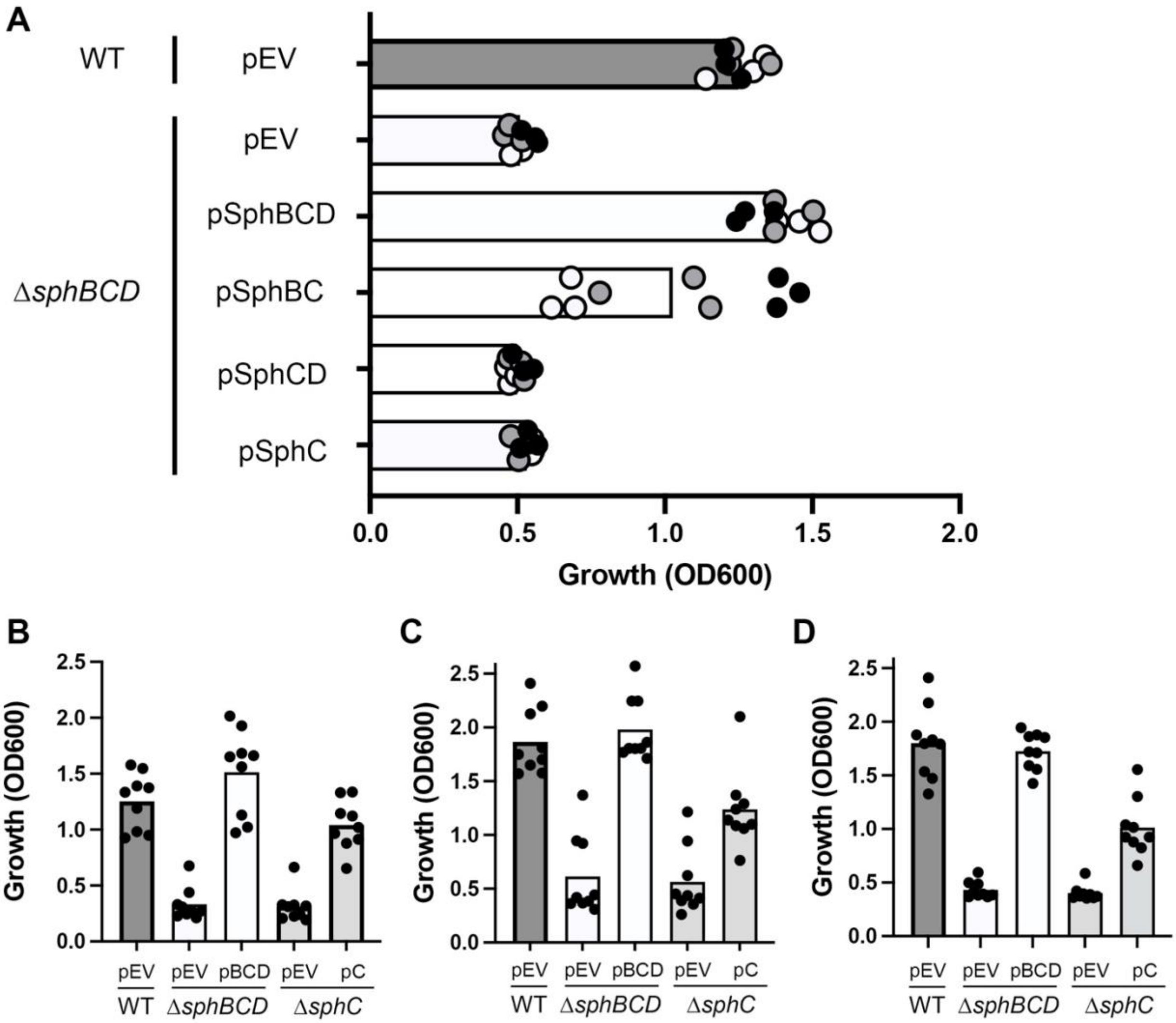

Supplemental Figure S4. The *sphBC* genes are critical for wild-type levels of growth in the presence of 200 μM sphingoid bases. **(A)** Complementation analysis of Δ*sphBCD* shows significant complementation only with plasmids expressing *sphB* and *sphC*, while *sphD* appears dispensable for growth (OD600) at 18 hour **(B-D)** Deletion of *sphC* phenocopies deletion of *sphBCD* and can be complemented by *sphC* on a plasmid. This phenotype is shared between the sphingoid bases **(B)** sphingosine, **(C)** sphinganine, and **(D)** phytosphingosine. Abbreviations: pEV, empty vector pMQ80; pBCD, vector containing *sphBCD;* pC, vector containing *sphC*.

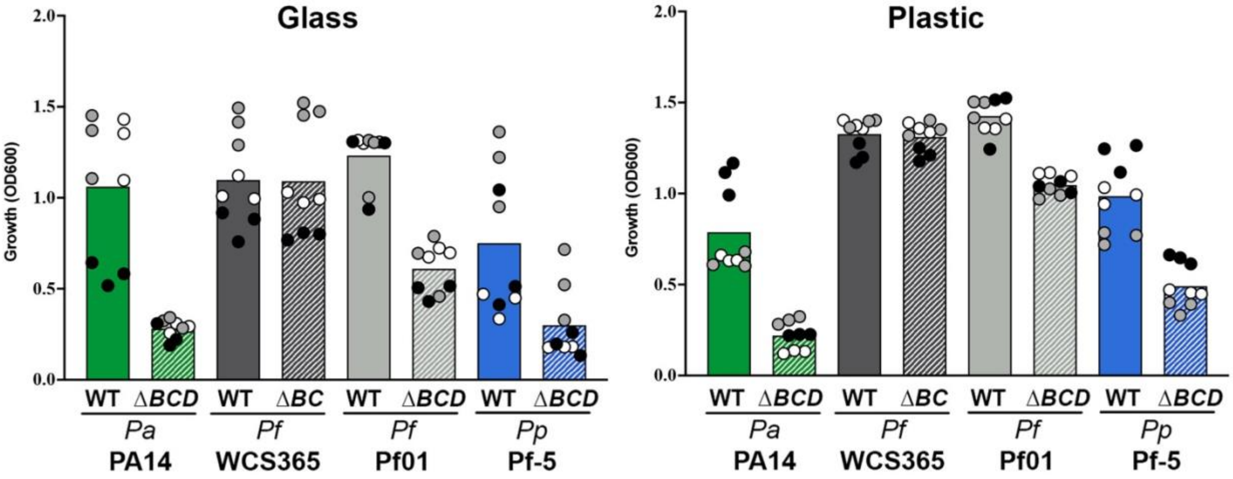

Supplemental Figure S5. The role of *sphBC* in other Pseudomonads. The growth (OD600) for WT and mutant for each strain in 200 µM sphingosine in MOPS media with 20 mM pyruvate is shown. As seen for *P. aeruginosa* PAO1 (Fig 2), the culture vessel material impacts the potency of sphingosine for some strains. For both panels, all data points are shown and are colored by experiment with white circles for all replicates from experiment #1, gray from experiment #2, and black from experiment #3. Abbreviations: ΔBCD, Δ*sphBCD*; ΔBC, Δ*sphBC*; Pa, *P. aeruginosa*; Pf, *P. fluorescens*; Pp, *P. protogens*.

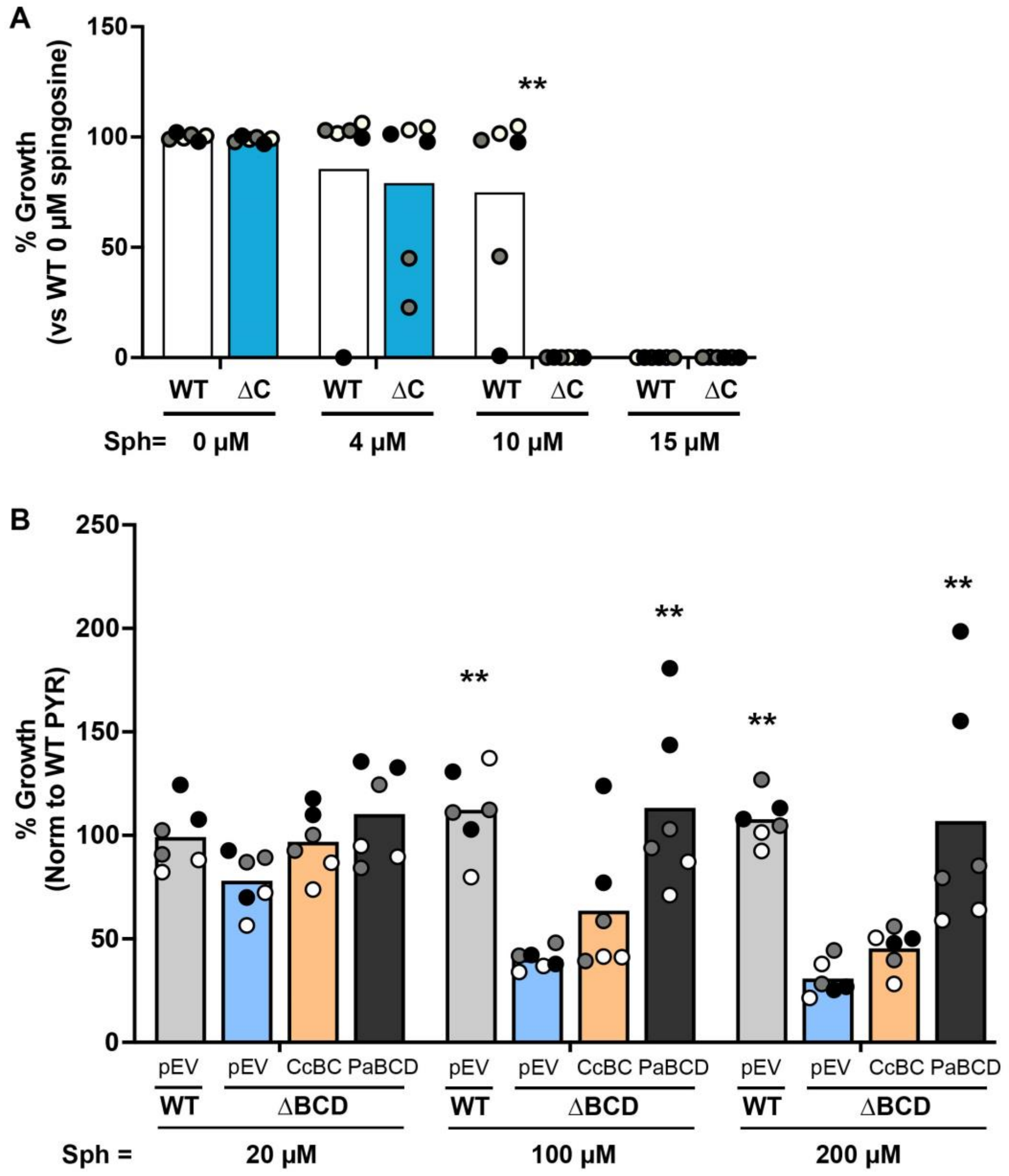

Supplemental Figure S6: Tests of *Caulobacter crescentus sphBC* function. (A) Deletion of the *Caulobacter crescentus sphC* has very little impact on growth in the presence of sphingosine after 18-hour growth in glass when considering the small concentration range of sphingosine over which the effect is noted. **(B)** Complementation analyses of *P. aeruginosa* Δ*sphBCD* with empty vector (pEV), a plasmid containing *C. crescentus sphBC* (CcBC), or a plasmid containing *P. aeruginosa sphBCD* (PaBCD) shows that *C. crescentus sphBC* fails to significantly complement the *P. aeruginosa* Δ*sphBCD* mutant. All data points are shown and are colored by experiment with white circles for replicates from experiment #1, gray from experiment #2, and black from experiment #3. Only the means for each experiment are used in the statistical analyses for these panels (n = 3 per condition). In **A**, significance noted as (**, p<0.01) calculated using multiple Mann-Whitney tests comparing WT to Δ*sphC* within each sphingosine concentration. This test was chosen since the zero growth as a mean within an experiment make the data non-parametric. In **B**, significance noted as (**, p<0.01) calculated from Two-way ANOVA with Dunnett's post-test comparing each group to the Δ*sphBCD* + pEV group within each concentration. Abbreviations: Sph, sphingosine; Pyr, pyruvate.

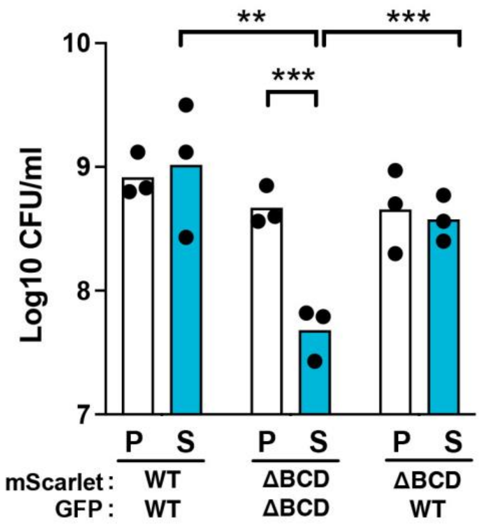

Supplemental Figure S7: Swap of the fluorescent marker as used in Figure 7. CFU counts of mScarlet-expressing colonies in the presence (S) or absence (P) of sphingosine. The strain carrying each fluorescent protein is labeled below graph. Significance noted as (**, p<0.01; ***, p<0.001) calculated from ANOVA with Tukey's post-test comparing within and between co-culture groups. Each point represents the mean from a single experiment (n = 3 per condition). Abbreviations: ∆BCD, ∆*sphBCD*; P, pyruvate (control); S, sphingosine; N.D., not detectable.

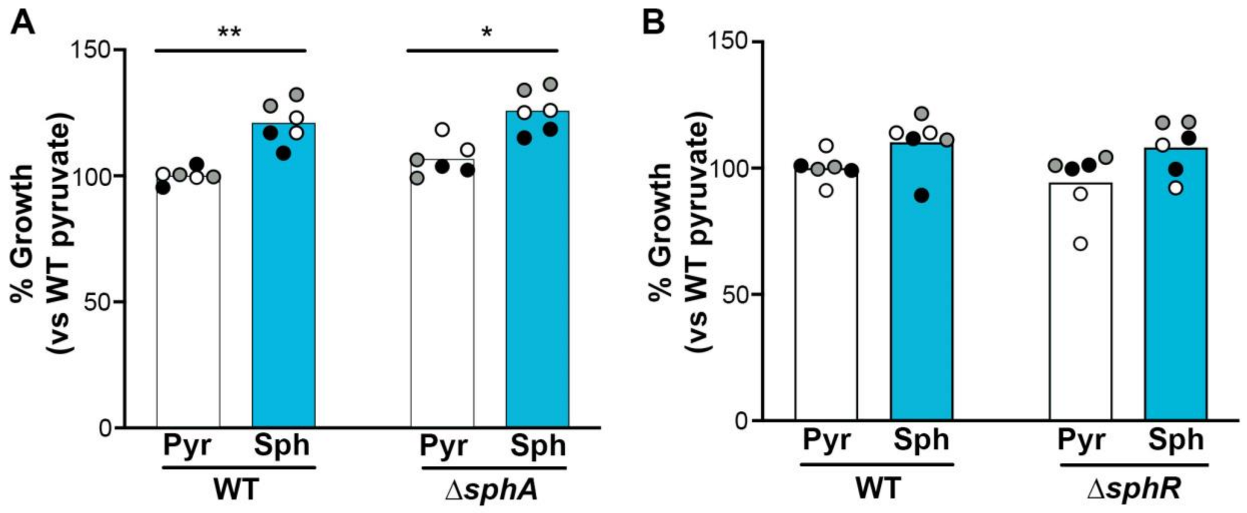

Supplemental Figure S8: Effects of *sphA* and *sphR* deletion on sphingosine susceptibility. 18-hour growth was normalized to WT growth in MOPS pyruvate set as 100%. **(A)** PAO1 WT compared to the *sphA* deletion strain. **(B)** PAO1 WT compared to the *sphR* deletion strain. For each panel, all data points are shown and are colored by experiment with white circles for replicates from experiment #1, gray from experiment #2, and black from experiment #3. Only the means for each experiment are used in the statistical analyses for these panels (n = 3 per condition). Significance noted as (*, $p<0.05$; **, $p<0.01$) calculated from Two-way ANOVA with Sidak's post-test comparing pyruvate to sphingosine within each strain. Abbreviations: Pyr, pyruvate; Sph, sphingosine.

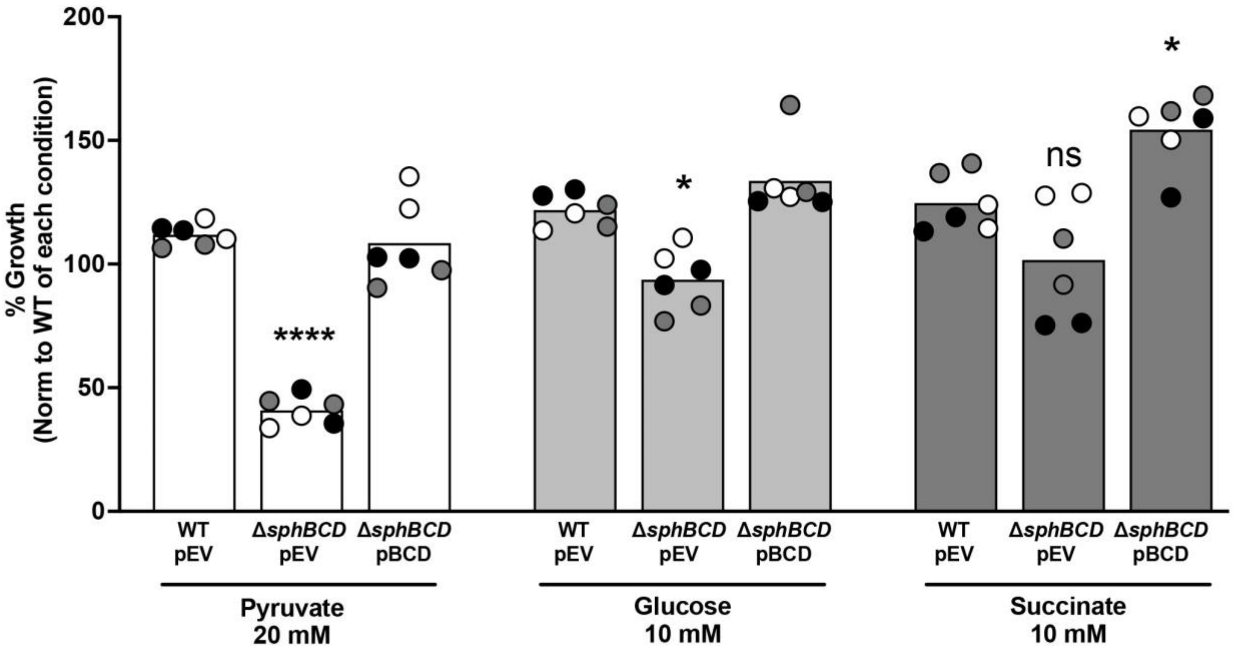

Supplemental Figure S9: Effect of carbon source on sphingosine inhibition and the role of *sphBCD*. 18-hour growth was normalized to WT+pEV growth in MOPS pyruvate set as 100%. All data points are shown and are colored by experiment with white circles for replicates from experiment #1, gray from experiment #2, and black from experiment #3. Only the means for each experiment are used in the statistical analyses for these panels (n = 3 per condition). Significance noted as (*, $p<0.05$; ****, $p<0.0001$) calculated from Two-way ANOVA with Dunnett's post-test comparing deletion or complement within each carbon source condition to its WT+pEV. Abbreviations: pEV, empty vector pMQ80; pBCD.

Supplemental Table S1: Primers used in this study

Primer ID	Primer Name	Primer Sequence
2080	PAsphBCD_KO_F1_KpnI	AGG GTA CCA TGG AAA ACC ACG ACA CCG ACT AT
2082	PAsphBCD_KO_R2_HindIII	AGG AAG CTT GCT GGC TCT GTC GCT CGT TCG CAT
2081	PAsphBCD_KO_F2_SOE_SmaI	CCC GGG ATG CTC AAG CCG AGC CAC TAC GAC CTG GCG
2083	PAsphBCD_KO_R1_SOE_SmaI	CGG CTT GAG CAT CCC GGG GGT GTT CCT CTC TCG TTG
1022	sphC 5327 KO A Forward HindIII	AAG CTT TAT TCC GCC AGT TGC AAG CTC TGT
1024	sphC 5327 KO B Forward SOE	CCA TGG TCG AGT CGC CTT CGT ACT TAA TCG ACG CAA GTT CAT GTT CGC C
1023	sphC 5327 KO A Reverse SOE	AAG TAC GAA GGC GAC TCG ACC ATG GAC CAG TTG CGC CAG GGA ATC A
1025	sphC 5327 KO B Reverse BamHI	GGA TCC TTG AGG AAC GGT TGG TGG AAG GA
2736	PfWSC365_04425-30_F1_XbaI	AATTCTAGAACACTAAGGCACGCCACTG
2737	PfWSC365_04425-30_R1_KpnI	AATGGTACCTAGATCATGCCGTTGATGGA
2738	PfWSC365_04425-30_F2_HindIII	AATAAGCTTCATAGGTCGGGTCGATGACT
2739	PfWSC365_04425-30_R2_XbaI	AATTCTAGATTCCCATCGAATACCGCTAC
2740	PF01_RS12590-12600_F1_XbaI	AATTCTAGAAGCCCATGCAGATAATCGAC
2741	PF01_RS12590-12600_R1_BamHI	AATGGATCCAGTCGGGTTCCCTGAAGAAT
2742	PF01_RS12590-12600_F2_BamHI	AATGGATCCCTGGCCGGTGTATACCTGAT
2743	PF01_RS12590-12600_R2_KpnI	AATGGTACCCCCAACTGCCAAAGATTGTT
2732	Pf5sphBCD_F1_KpnI	AATGGTACCCGGTACTGACCACCCAACTG
2733	Pf5sphBCD_R1_XbaI	AATTCTAGACGAATCGGTAGCCAGGAGTG
2734	Pf5sphBCD_F2_XbaI	AATTCTAGAGCGCCAGACCTTCTTCATCT
2735	Pf5sphBCD_R2_HindIII	AATAAGCTTACTTCACCACCTACAAGCCG
2726	PAO1sphBCDcompFEcoRI	AATGAATTCGAAGGTGTAGTTCTGGCGCT
2727	PAO1sphBCDcompRHindIII	AATAAGCTTCTCTGAGGCATCGGAACGAA
2511	sphC-exp-F EcoRI	CAAGGAATTCCCGCAGCGCAGGACCGATAGGGGA
2512	sphC-exp-R-untagged-HindIII	CAGAAAGCTTCTAGGTCACGCCCAGGATGGAAGA
2744	PAO1_sphC_RTF	GTAGTGCTGATGCACGGAAA
2745	PAO1_sphC_RTR	GATTCCTATGCGGTCTACGC
2882	sphBC-comp-F1	CGTTGTAAAACGACGGCCAGTGCCAGTAGGGTACCCATCGACTCTAGACCGCCAT
2883	sphBC-comp-R1	GACCATGATTACGAATTCGAGCTCGAAGCTTGTCCAGGCGATCGAGGT
2598	sphBdel_R_SOE	GCAGCGGCAGCGGATTTCATGTCCGCA
2599	sphBdel_F_SOE	CGCTGCCGCTGCGCAAAGGCGCGAGTT
587	sphD-RT-F	CATCCGCAGTACCTGGAGTT
588	sphD-RT-R	CTGCTCAGCGTTTCCTTCTC
1372	89GFP GFP F	TGCGCAACCTCAACCCCGGAGCGCCCCATGAGTAAAGGAGAAGAACTTTTCACT
1373	89GFP GFP R	TAGGTACCTAACTATTTGTATAGTTCATCCAT
